# Variations in use of childbirth interventions in 13 high-income countries: A multinational cross-sectional study

**DOI:** 10.1371/journal.pmed.1003103

**Published:** 2020-05-22

**Authors:** Anna E. Seijmonsbergen-Schermers, Thomas van den Akker, Eva Rydahl, Katrien Beeckman, Annick Bogaerts, Lorena Binfa, Lucy Frith, Mechthild M. Gross, Björn Misselwitz, Berglind Hálfdánsdóttir, Deirdre Daly, Paul Corcoran, Jean Calleja-Agius, Neville Calleja, Miriam Gatt, Anne Britt Vika Nilsen, Eugene Declercq, Mika Gissler, Anna Heino, Helena Lindgren, Ank de Jonge

**Affiliations:** 1 Department of Midwifery Science, AVAG, Amsterdam Public Health research institute, Amsterdam UMC, location VUmc, Amsterdam, the Netherlands; 2 Department of Obstetrics, Leiden University Medical Center, Leiden, the Netherlands; 3 Athena Institute, Vrije Universiteit Amsterdam, Amsterdam, the Netherlands; 4 University College Copenhagen, Department of Midwifery, Copenhagen NV, Denmark; 5 Nursing and Midwifery Research unit, faculty of Medicine and Pharmacy, Vrije Universiteit Brussel, Brussels, Belgium; 6 Department of Development and Regeneration KU Leuven, University of Leuven, Leuven, Belgium; 7 Faculty of Medicine and Health Sciences, Centre for Research and Innovation in Care (CRIC), University of Antwerp, Belgium; 8 Department of Women´s and Newborn Health Promotion-School of Midwifery, Faculty of Medicine, University of Chile, Santiago, Chile; 9 Department of Health Services Research, The University of Liverpool, Liverpool, United Kingdom; 10 Midwifery Research and Education Unit, Department of Obstetrics, Gynaecology and Reproductive Medicine, Hannover Medical School, Hannover, Germany; 11 Institute of Quality Assurance Hesse, Eschborn, Germany; 12 Midwifery Programme, Faculty of Nursing, School of Health Sciences, University of Iceland, Reykjavík, Iceland; 13 School of Nursing and Midwifery, Trinity College Dublin, Dublin, Ireland; 14 National Perinatal Epidemiology Centre, Department of Obstetrics and Gynaecology, University College Cork, Cork, Ireland; 15 Department of Anatomy, Faculty of Medicine and Surgery, University of Malta, Tal-Qroqq, Msida, Malta; 16 Directorate for Health Information and Research, Gwardamangia, Malta; 17 Department of Public Health Department, Faculty of Medicine and Surgery, University of Malta, Tal-Qroqq, Msida, Malta; 18 Western Norway University of Applied Sciences (HVL), Department of Health and Caring Sciences, Bergen, Norway; 19 Boston University School of Public Health, Boston, United States of America; 20 THL Finnish Institute for Health and Welfare, Information Services Department, Helsinki, Finland; 21 Karolinska Institute, Department of Neurobiology, Care Sciences and Society, Huddinge, Sweden; 22 Department of Women’s and Children’s Health, Karolinska Institutet, Solna, Sweden; University of Edinburgh, UNITED KINGDOM

## Abstract

**Background:**

Variations in intervention rates, without subsequent reductions in adverse outcomes, can indicate overuse. We studied variations in and associations between commonly used childbirth interventions and adverse outcomes, adjusted for population characteristics.

**Methods and findings:**

In this multinational cross-sectional study, existing data on 4,729,307 singleton births at ≥37 weeks in 2013 from Finland, Sweden, Norway, Denmark, Iceland, Ireland, England, the Netherlands, Belgium, Germany (Hesse), Malta, the United States, and Chile were used to describe variations in childbirth interventions and outcomes. Numbers of births ranged from 3,987 for Iceland to 3,500,397 for the USA. Crude data were analysed in the Netherlands, or analysed data were shared with the principal investigator. Strict variable definitions were used and information on data quality was collected. Intervention rates were described for each country and stratified by parity. Uni- and multivariable analyses were performed, adjusted for population characteristics, and associations between rates of interventions, population characteristics, and outcomes were assessed using Spearman’s rank correlation coefficients. Considerable intercountry variations were found for all interventions, despite adjustments for population characteristics. Adjustments for ethnicity and body mass index changed odds ratios for augmentation of labour and episiotomy. Largest variations were found for augmentation of labour, pain relief, episiotomy, instrumental birth, and cesarean section (CS). Percentages of births at ≥42 weeks varied from 0.1% to 6.7%. Rates among nulliparous versus multiparous women varied from 56% to 80% versus 51% to 82% for spontaneous onset of labour; 14% to 36% versus 8% to 28% for induction of labour; 3% to 13% versus 7% to 26% for prelabour CS; 16% to 48% versus 12% to 50% for overall CS; 22% to 71% versus 7% to 38% for augmentation of labour; 50% to 93% versus 25% to 86% for any intrapartum pain relief, 19% to 83% versus 10% to 64% for epidural anaesthesia; 6% to 68% versus 2% to 30% for episiotomy in vaginal births; 3% to 30% versus 1% to 7% for instrumental vaginal births; and 42% to 70% versus 50% to 84% for spontaneous vaginal births. Countries with higher rates of births at ≥42 weeks had higher rates of births with a spontaneous onset (rho = 0.82 for nulliparous/rho = 0.83 for multiparous women) and instrumental (rho = 0.67) and spontaneous (rho = 0.66) vaginal births among multiparous women and lower rates of induction of labour (rho = −0.71/−0.66), prelabour CS (rho = −0.61/−0.65), overall CS (rho = −0.61/−0.67), and episiotomy (multiparous: rho = −0.67). Variation in CS rates was mainly due to prelabour CS (rho = 0.96). Countries with higher rates of births with a spontaneous onset had lower rates of emergency CS (nulliparous: rho = −0.62) and higher rates of spontaneous vaginal births (multiparous: rho = 0.70). Prelabour and emergency CS were positively correlated (nulliparous: rho = 0.74). Higher rates of obstetric anal sphincter injury following vaginal birth were found in countries with higher rates of spontaneous birth (nulliparous: rho = 0.65). In countries with higher rates of epidural anaesthesia (nulliparous) and spontaneous births (multiparous), higher rates of Apgar score < 7 were found (rhos = 0.64). No statistically significant variation was found for perinatal mortality. Main limitations were varying quality of data and missing information.

**Conclusions:**

Considerable intercountry variations were found for all interventions, even after adjusting for population characteristics, indicating overuse of interventions in some countries. Multivariable analyses are essential when comparing intercountry rates. Implementation of evidence-based guidelines is crucial in optimising intervention use and improving quality of maternity care worldwide.

## Abstract 2

Anna Seijmonsbergen-Schermers and co-authors report on use of childbirth interventions and associated outcomes in high-income countries.

## Author summary

**Why was this study done?:**

- Interventions during childbirth are important to prevent adverse outcomes in mothers and children. However, large variations in childbirth interventions are indicators of over- or underuse.
- Variations in childbirth interventions rates have been studied before, but these studies did not account for differences in population characteristics, such as maternal age and body mass index.
- We conducted this study, including data of 13 countries, to describe variations adjusted for population characteristics; to examine correlations between interventions and between interventions and adverse outcomes; and to describe the quality of data.

**What did the researchers do and find?:**

- We included data from 4,729,307 singleton births at ≥37 weeks in 13 countries in 2013.
- We found large variations in the use of childbirth interventions between these countries, without a significant difference in perinatal mortality rates. Largest variations were found for augmentation of labour, pain relief, episiotomy, instrumental vaginal birth, and cesarean section.
- Variations remained after adjustments for differences in population characteristics.
- We found several correlations between interventions and a few correlations between interventions and adverse outcomes. For instance, countries with higher rates of prelabour cesarean section had also higher rates of emergency cesarean section.
- Quality of data, methods of data collection, and definition of variables varied across countries.

**What do these findings mean?:**

- The findings suggest that some childbirth interventions are frequently overused in many countries. Quality of maternity care needs to be improved, for instance, through implementation of international guidelines.
- Adjusting for population characteristics is important in order to make valid comparisons between countries.
- The findings on quality of the data warrant improvement of data quality, including uniformly recorded country-level data being freely available for research.

## Introduction

Interventions during childbirth can be necessary to prevent maternal and perinatal mortality and morbidity [1]. The use of many interventions during childbirth is increasing worldwide and can improve maternal and perinatal outcomes in facilities where the care provided is too little or too late [2,3]. In contrast, some interventions are performed too frequently and too soon in several countries [2]. Although interventions have potential advantages, they are not without risks that may harm women, physically and mentally, and their babies, and therefore, unnecessary use should be avoided [4]. Furthermore, overuse leads to unnecessarily high healthcare costs [5]. The use of interventions should be a balance between being sufficient to improve maternal and perinatal outcomes and not exceeding the ‘point of optimality’, after which more frequent use will lead to more harm than benefit at population level [6]. A certain amount of intercountry variation in the use of childbirth interventions is expected and may be necessary, because maternal and perinatal characteristics differ between countries and this may affect clinical decision-making. Conversely, unexpectedly large variations in the use of childbirth interventions without improved outcomes in areas with high use are important indicators of overuse [4]. Wide variations are unexpected when not attributable to differences in population characteristics [4].

Several previous international studies and reports [7–10] have described variations in intervention rates, but these were limited by the lack of adjustments for population characteristics. This study sought to explore correlations between interventions and births without interventions and whether higher or lower intervention rates were accompanied by higher or lower rates of adverse maternal and/or perinatal outcomes. The aim of our study was 3-fold: first, to describe the range of variations in commonly used childbirth interventions, and birth outcomes, among nulliparous and multiparous women in high-income countries around the world, by comparing rates, adjusted for population characteristics; second, to examine correlations between interventions and between interventions and adverse maternal and neonatal outcomes; third, to describe the quality of the data from the respective countries.

## Methods

### Data collection

The study methods have been described previously in a prospective study protocol [11]. This study is reported according to the Strengthening the Reporting of Observational Studies in Epidemiology (STROBE) guideline (S1 STROBE Checklist). In summary, existing data were obtained, mainly, from national registry data from high-income countries for the year of birth of 2013. This multinational cross-sectional study was conceived during a meeting of European Cooperation in Science and Technology (COST) Action IS1405 ‘BIRTH’ [12]. All countries included in the COST Action were asked whether they had access to national data or data from a representative sample and whether they were interested in taking part in this study. Investigators in 27 countries expressed interest in taking part, and of these countries, we explored whether the data met the inclusion criteria. Countries were included based on availability of data, permission of data usage, and whether the a priori inclusion criteria could be met. Inclusion criteria were availability of anonymised data from a representative sample of all births of a singleton from 37 weeks’ gestation onwards in a country or state and available data on parity, gestational age at birth, and singleton or multiple gestation. Twelve of the 27 countries invited and one state of a country were able to provide sufficient data for inclusion in the study (Finland, Sweden, Norway, Denmark, Iceland, Ireland, England, the Netherlands, Belgium, the state of Hesse [Germany], Malta, USA, and Chile). Data from eight countries (Norway, Iceland, Ireland, the Netherlands, Belgium, Malta, USA, and Chile) were analysed in the Netherlands, because anonymised crude data from these countries were available. Data from four other countries and one state within a country (Finland, Sweden, Denmark, England, and the state of Hesse [Germany]) were analysed in the country itself, in close collaboration with the principal investigator in the Netherlands, because transfer of crude data was not possible due to data protection or ethical regulations or the time required to get a data transfer approval.

Participation of the other 14 invited countries was not possible. In some countries, the costs of preparing the data for transfer were too high or the procedures to retrieve the required data proved too complicated. In other countries, the available data were unsuitable because they were not from a representative sample of the population or were largely incomplete.

### Independent and dependent variables

The following variables were defined as population characteristics: parity (nulliparous; multiparous), maternal age (<20; 20–24; 25–29; 30–34; 35–39; ≥40 years), maternal body mass index (BMI) (<18.5; 18.5–24.9; 25.0–29.9; 30.0–34.9; 35.0–39.9; ≥40.0 kg/m^2^), gestational weight gain (mean IQR, in grams), ethnicity (majority group; minorities), education (high; medium; low), and socioeconomic status (high; medium; low). It was not possible to categorise ethnicity in native versus nonnative populations, because of difficulties in defining these groups, particularly for the USA. Therefore, we categorised this variable as ‘majority group’ or ‘minorities’. For the USA, additional categories were added to clarify allocation of ethnicity because a distinction was made between non-Hispanic and Hispanic ethnicity, with additional subgroups within these ethnic groups. Because socioeconomic status was not recorded in most countries, the variable ‘education’ was added. Education was categorised as high (bachelor degree or postsecondary-level education), medium (secondary or high school level), and low (primary school, without finishing secondary school, or no education). These categories were based on consensus of the authors in order to be able to combine the categories of all countries reporting educational level. The following variables were defined as birth characteristics: gestational age (37; 38; 39; 40; 41; ≥42 weeks), birth weight (<2,500; 2,500–3,499; 3,500–4,499; ≥4,500 g), place of birth (hospital; birth centre; home), and responsible care provider at onset of labour and at birth (midwife/nurse midwife; obstetrician/gynaecologist; general practitioner; other).

Primary outcomes added after publication of the study protocol [11] were onset of labour (spontaneous; induction of labour; prelabour cesarean section [CS]), intrapartum use of oxytocin, intrapartum antibiotics, and any pharmacological intrapartum pain relief. The variable ‘onset of labour’ was adjusted with respect to induction of labour and prelabour CS. Intrapartum use of oxytocin included either use of oxytocin for induction of labour or for augmentation of labour. The type of CS was defined based on the time of the intervention. A prelabour CS was a CS before the active phase of labour and includes emergency CS during pregnancy. A birth could be allocated to either induction or prelabour CS. A CS after induction of labour was defined as emergency CS during labour. Any intrapartum pharmacological pain relief included either epidural anaesthesia or other methods of pharmacological pain relief (e.g., pethidine injections). Pharmacological pain relief was defined as the use of medication to relieve labour pain, and therefore, prelabour CS was excluded from analyses. However, it was not possible for all countries to discern epidural/spinal anaesthesia for emergency CS performed after the onset of labour from epidural/spinal anaesthesia for labour pain. The term used to describe CS was changed from ‘planned versus emergency’ to ‘prelabour versus emergency during labour’ because in most countries, CS was categorised as prelabour or during labour. However, in some countries, the distinction was based on whether the CS was in fact a clinical emergency. When the reason for the CS was described as a clinical emergency but the exact timing of CS was not recorded, e.g., before or during labour, this CS was assigned to the category of ‘emergency during labour’. Augmentation of labour was described for births with a spontaneous onset, pain medication for births without prelabour CS, episiotomy and obstetric anal sphincter injury (OASI) for vaginal births (excluding CSs), and Apgar score < 7 after 5 minutes for live births. OASI was defined as a perineal tear with a disruption of the anal sphincter muscles and/or to the anal epithelium [13]. Other primary outcomes were artificial rupture of membranes, spontaneous vaginal birth, instrumental vaginal birth, active management of third stage of labour, and use of oxytocin postpartum. Adverse maternal and neonatal outcomes were perinatal mortality up to 7 days, Apgar score below 7 at 5 minutes, maternal mortality up to 7 days, postpartum haemorrhage ≥ 1,000 ml (PPH), and OASI.

### Analysis and missing data

Results were analysed and presented by country, and total numbers in each country and percentages of each variable were presented. Intervention rates were stratified for nulliparous and multiparous women. For each variable, the percentages of missing data were calculated. Univariable analyses were conducted on variations in rates of interventions and adverse outcomes, and multivariable analyses were conducted to adjust these variations for parity, maternal age, ethnicity, prepregnancy BMI, education, and infant birth weight. Unadjusted and adjusted odds ratios (aORs) were calculated, with the mean incidence of the variable as the reference category, weighted for the sample size of each country to ensure that all countries contributed equally. A 99% confidence interval (CI) was used to account for multiple testing in a large data set. The eight countries that provided crude data were included in the multivariable logistic regression analyses. Based on the study protocol, if more than 10% of data on a dependent variable were missing for a country, this country’s data were excluded from the analysis for that specific variable. Data on an independent variable with more than 5% of missing data were included in the multivariable analyses using multiple imputation. Data for the variables ethnicity, BMI, and education were imputed for Norway, Iceland, the Netherlands, Belgium, Malta, and Chile, using predictive mean matching in the R package Multivariate Imputation via Chained Equations (mice) for each country separately [14], and for the USA in STATA version 14. Multiple imputed data from the USA were too large to be included in the multivariable analyses. Therefore, data from USA were randomly compressed 10 times in multivariable analyses including ethnicity and education.

As described in the protocol [11], we linked the results of the interventions to obtain better understanding of relationships between interventions and presented these correlations in graphs. However, we also calculated Spearman’s rank correlation coefficients to quantify the intercountry associations of intervention rates, population characteristics, and adverse outcomes. Spearman’s rank correlation coefficients were calculated on variables when data from at least eight countries were available and only if the unadjusted ORs were statistically significantly different across countries. A correlation of −0.60 ≤ rho ≥ 0.60 was considered strong [15] and indicated in bold text in the tables.

Statistical analyses were performed using STATA version 14 (StataCorp, Texas, USA) and R Statistics 3.6.0.

### Quality assessment

Data quality was assessed using five questions on standard quality procedures, missing data, dealing with contradictions, data coverage, and other data checks performed in each country [16]. When crude data were used, the same definitions of variables were used for all countries, and the analyses were conducted by the principal investigator in the Netherlands. When aggregate data were provided by a country, these were analysed by the investigator of the country in close collaboration with the principal investigator in the Netherlands who provided detailed instructions on methods and variables.

### Ethics approval

Ethical approval was not needed for all countries. This study has been approved by the Amsterdam UMC, location VUmc (reference WC2016-055; http://www.ccmo.nl/en/your-research-does-it-fall-under-the-wmo); the Regional Committees for Ethics in Medical Research in Norway (reference 2017/616/REK vest); and the National Bioethics Committee in Iceland (reference VSN-16-157).

## Results

### Included countries and missing data

Data from the following countries were included in this study: Finland, Sweden, Norway, Denmark, Iceland, Ireland, England, the Netherlands, Belgium, the state of Hesse (Germany), Malta, USA, and Chile. The total number of births in this study was 4,729,307. National characteristics, proportions of the total number of births included for each country, and data sources used can be found in S1 Table. In one country, two dependent variables (‘artificial rupture of membranes’ and ‘use of oxytocin postpartum’) had more than 10% missing and these were excluded from further analyses. Missing population characteristics concerning BMI and education were not randomly distributed. Missing data in the independent variables ethnicity, BMI, and education were imputed for use in the multivariable analyses because of missing data in more than 5% of the records.

### Variations in population and birth characteristics

Population and birth characteristics are presented in Tables 1 and 2. Rates of births at ≥42 weeks’ gestation varied from 0.1% in Malta and Chile to 6.7% in Sweden. Countries with the lowest numbers of births at ≥42 weeks’ gestation had higher numbers of births at 37 weeks’ gestation (varying from 4.2% in Iceland to 9.1% in Chile) and vice versa. We found a similar pattern for infant birth weight: countries with lower rates of birth weight < 2,500 g had higher rates of birth weight ≥ 4,500 g and vice versa (Table 2). The proportion of births in a hospital or birth centre varied from 85.1% in the Netherlands to 99.98% in Chile, with lower rates of epidural anaesthesia (rho = 0.62) among nulliparous women in countries with higher rates of out-of-hospital births.

**Table 1:** Population characteristics by country of births of a single child at ≥37 weeks’ gestation in 2013.

Characteristics	FIN	SWE	NOR	DNK	ISL	IRL	ENG	NLD	BEL	HESSE	MLT	USA	CHL
**Parity, %**													
Nulliparous women	40.4	43.8	41.6	46.4	39.6	37.9	39.9	44.7	43.2	50.0	50.9	39.8	44.0
Multiparous women	59.6	56.2	58.4	53.6	60.4	62.1	60.1	55.3	56.9	50.0	49.1	60.2	56.0
Missing, %	0.0	0.2	0.0	0.12	0.0	0.03	21.2	0.0	0.0	0.0	0.0	0.0	0.0
**Total *n***	5,431	10,457	54,951	50,692	3,987	62,592	410,366	152,644	112,907	44,722	3,781	3,500,397	173,388
**Maternal age, %**													
<20 years	2.0	14.8*	1.5	1.4	1.9	2.1	4.8	1.2	1.9	1.8	4.0	7.1	14.2
20–24 years	14.7		13.9	11.3	17.1	9.6	18.8	10.0	13.1	11.6	13.2	23.0	24.6
25–29 years	30.5	31.2	32.2	31.1	30.6	20.7	28.7	30.8	34.4	26.9	30.5	28.8	24.0
30–34 years	33.2	33.6	33.5	35.2	30.6	36.9	28.9	37.6	33.7	34.9	35.7	26.4	21.2
35–39 years	16.3	20.4*	15.8	17.5	15.8	25.3	14.8	17.2	13.9	20.1	14.5	12.0	12.3
≥40 years	3.5		3.2	3.6	4.0	5.4	3.6	3.2	3.0	4.7	2.1	2.8	3.7
Missing, %	0.0	0.1	0.01	0.0	0.0	0.0	1.2	<0.01	0.0	0.0	0.0	0.0	0.0
**Maternal body mass index, %**													
<18.5 kg/m^2^	3.5	2.8	4.2	4.4	3.0	-	-	-	5.4	3.7	2.8	3.9	-
18.5–24.9 kg/m^2^	61.0	56.8	61.1	62.0	55.5	-	-	-	60.1	58.7	58.8	47.3	-
25.0–29.9 kg/m^2^	22.3	24.5	22.3	21.1	23.7	-	-	-	22.2	23.5	23.8	25.4	-
30.0–34.9 kg/m^2^	8.8	12.3*	8.6	8.3	11.2	-	-	-	8.6	9.0	9.5	13.1	-
35.0–39.9 kg/m^2^	3.1		2.8	2.9	4.3	-	-	-	2.7	3.4	3.7	6.2	-
≥40.0 kg/m^2^	1.3		1.1	1.4	2.4	-	-	-	0.9	1.7	1.3	4.2	-
Missing, %	1.3	3.7	36.1	1.2	19.3				5.4	8.0	22.9	13.5	
**Gestational weight gain, mean (IQR), in kilograms**	-	10.6	14.1	-	14.4	-	-	-	12.5	13.7	14.5	13.7	-
		(7–15)	(10–18)		(11–18)				(9–16)	(10–17)	(11–18)	(10–18)	
Missing, %		-	71.5		30.6				7.6	13.9	67.3	13.9	
**Ethnicity, %**													
Majority group	90.3	73.0	72.9	81.8	88.2	-	79.6	75.1	78.9	79.2	86.5	76.5^$^	-
Minorities	9.7	27.0	27.1	18.2	11.8	-	20.4	24.9	21.1	20.8	13.5	23.5	-
Missing, %	0.5	0.0	1.4	1.7	0.1		7.2	0.8	8.7	0.0	0.2	0.0	
Non-Hispanic:													
White	-	-	-	-	-	-	-	-	-	-	-	57.9	-
Black	-	-	-	-	-	-	-	-	-	-	-	14.6	-
American Indian or Alaskan Native	-	-	-	-	-	-	-	-	-	-	-	1.0	-
Asian/Pacific Islander	-	-	-	-	-	-	-	-	-	-	-	6.6	-
Hispanic	-	-	-	-	-	-	-	-	-	-	-	19.8	-
Missing, %												0.0	
**Education, %**													
High	45.2	52.3	-	43.7	-	-	-	-	45.7	-	34.1	38.0	30.5
Medium	42.5	38.6	-	40.3	-	-	-	-	37.4	-	63.1	46.2	57.9
Low	14.2	9.1	-	16.6	-	-	-	-	16.8	-	2.8	15.8	11.6
Missing, %		12.0		8.7					10.8		3.9	10.5	0.01
**Socioeconomic status, %**													
High	19.2	-	-	-	-	-	35.0	21.2	-	-	-	-	-
Medium	37.9	-	-	-	-	-	45.6	45.4	-	-	-	-	-
Low	16.7	-	-	-	-	-	19.4	33.4	-	-	-	-	-
Missing, %	26.2						1.6	3.5					

*SWE: the age categories <20 years/20–24 years and 35–39 years/≥40 years and the body mass index categories of >30 kg/m^2^ are taken together in the Swedish data.

^$^USA: the majority group includes both non-Hispanic white women and Hispanic white women.

Abbreviations: BEL, Belgium; CHL, Chile; DNK, Denmark; ENG, England; FIN, Finland; HESSE, the state of Hesse (Germany); IRL, Ireland; ISL, Iceland; MLT, Malta; NLD, the Netherlands; NOR, Norway; SWE, Sweden; USA, United States of America

**Table 2:** Birth characteristics by country in 2013.

Characteristics	FIN	SWE	NOR	DNK	ISL	IRL	ENG	NLD	BEL	HESSE	MLT	USA	CHL
**Total *n***	54,310	104,570	54,951	50,692	3,987	62,613	410,366	152,644	112,907	44,722	3,781	3,500,397	173,477
**Gestational age, %^**													
37 weeks	4.6	-	5.0	4.7	4.2	-	6.1	7.6	7.7	7.2	8.5	8.7	9.1
38 weeks	13.2	-	12.5	13.7	11.8	-	13.7	16.6	20.1	20.2	23.3	17.8	31.3
39 weeks	27	-	25.1	23.4	27.8	-	24.8	26.6	30.7	27	32.2	42	31.6
40 weeks	30.2	-	30.4	30.7	32.3	-	30.1	29.7	30.6	30.1	32.3	24	22.9
41 weeks	20.3	-	23	25.7	21.7	-	20.9	18	10.8	14.9	3.5	7.1	5.1
≥42 weeks	4.7	6.7	4.0	1.9	2.2	-	4.4	1.4	0.3	0.6	0.1	0.5	0.1
**Birth weight, %**													
<2,500 g	1.0	2.1	1.0	1.1	0.5	-	2.5	1.8	2.2	1.6	2.8	2.4	1.7
2,500–3,499 g	96.3	43.1	42.8	44.5	35.9	-	53.2	49	58.7	54.8	65.5	58.9	57.2
3,500–4,499 g	53.5	51.3	53.1	51.6	58.8	-	42.3	47.2	38.2	42.4	31.2	37.5	40.5
≥4,500 g	2.7	3.5	3.2	2.7	4.8	-	1.9	2.1	0.9	1.2	0.6	1.2	0.6
Missing, %	0.02	0.03	0.0	1.0	0.0		2.3	0.1	0.03	0.0	0.0	0.1	0.0
**Place of birth, %**													
Hospital	99.7	98	99.1	99	94.3	-	84	85.1	99.3	98.4^$^	99.6	98.5	99.98
Birth centre	-	2.0	-	-	3.6	-	15.2	-	0.1	1.2	-	0.5	-
Home	0.04	0.01	0.2	0.8	2.0	-	- *	14.9	0.6	0.3	0.2	1.0	0.01
Other	0.3*	-	0.7*	0.2	0.2	-	0.9	-	0.01*	0.1	0.2*	0.1	0.01
Missing, %	0.0	0.0	0.0	0.7	0.0		1.0	0.8	0.01	0.0	0.0	<0.01	0.0
**Responsible care provider at onset of labour, %**													
Midwife	-	-	-	-	-	-	-	53.6	-	-	-	-	-
Obstetrician/gynaecologist	-	-	6.2^#^	-	-	-	-	46.1	-	-	-	-	-
Nurse midwife	-	-	93.8^#^	-	-	-	-	-	-	-	-	-	-
General practitioner	-	-	-	-	-	-	-	0.3	-	-	-	-	-
Missing, %			0.0					0.5					
**Responsible care provider at birth, %**													
Midwife/nurse midwife	-	74.5	74.8^#^	-	74.2^#^	-	58.0	30.1	0.7	-	-	9.6	-
Obstetrician/gynaecologist	-	25.5	25.2^#^	-	20.2	-	37.6	69.6	99.3	-	96	83.5	-
General practitioner	-	-	-	-	-	-	0.02	0.3	-	-	-	-	-
Other	-	-		-	5.5	-	4.3	-	-	-	4.0	6.9^#^	-
Missing, %		0.0	0.0		8.4		3.6	0.5	0.01		0.0	0.05	

*Place of birth:

• FIN: other place of birth: on the way to the hospital, unplanned births outside the hospital.

• NOR: ‘hospital’ includes small birth centres; other place of birth: during transport or unspecified place outside the institution (baby born before arrival).

• BEL: other place of birth: in a car, ambulance, on the way to the hospital.

• MLT: other place of birth: in a car, ambulance, on the way to the hospital, sometimes on a boat or at an emergency department.

• ENG: HIPE records hospital births only.

^$^HESSE: based on a total of 45,393 births. Only the births in the hospital or ‘other’ place are included in this study (*n* = 44,722), because a separate database with out-of-hospital births could not be linked.

^#^Care provider:

• NOR: at onset of labour: midwives are the responsible care provider in most of births without prelabour CS; at birth: midwives are the responsible care provider in most of spontaneous vaginal births (these numbers are based on the variables prelabour CS and spontaneous vaginal births).

• USA: other care provider: 6.2% of births are under responsibility of a doctor of osteopathy.

• CHL: in the data of Chile, it was only registered which care provider attended the birth. Although obstetricians are the last responsible care providers, midwives attend all normal births.

^CHL: birth is defined as birth from 24 weeks onwards, instead of 16 weeks.

Abbreviations: BEL, Belgium; CHL, Chile; CS, cesarean section; DNK, Denmark; ENG, England; FIN, Finland; HESSE, the state of Hesse (Germany); HIPE, Hospital In-Patient Enquiry; IRL, Ireland; ISL, Iceland; MLT, Malta; NLD, the Netherlands; NOR, Norway; SWE, Sweden; USA, United States of America

### Variations in intervention rates

**Fig 1:**
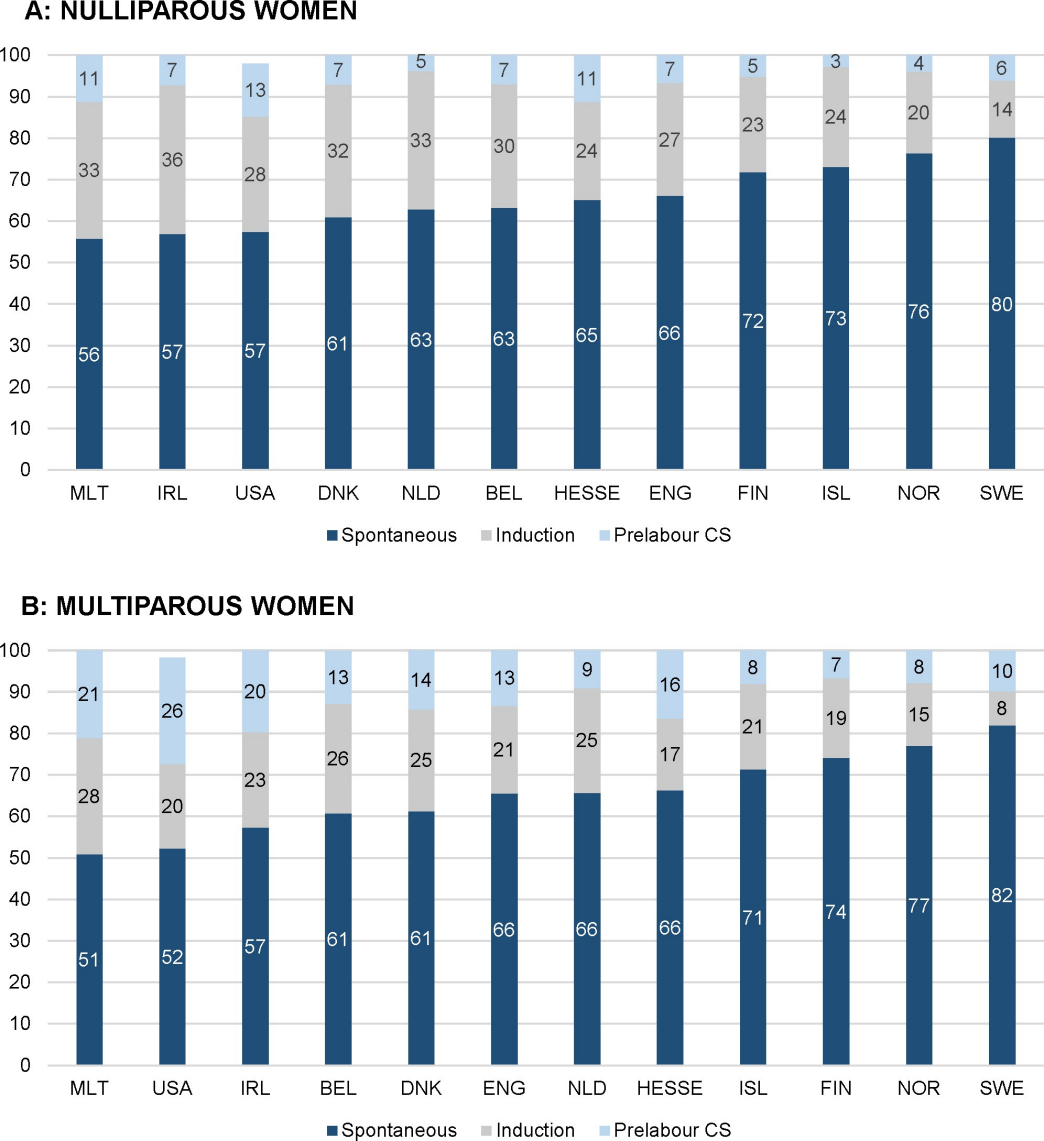
Intercountry variation of onset of labour in 2013 (%). (A) Nulliparous women. (B) Multiparous women. BEL, Belgium; CHL, Chile; CS, cesarean section; DNK, Denmark; ENG, England; FIN, Finland; HESSE, the state of Hesse (Germany); IRL, Ireland; ISL, Iceland; MLT, Malta; NLD, the Netherlands; NOR, Norway; SWE, Sweden; USA, United States of America.

**Fig 2:**
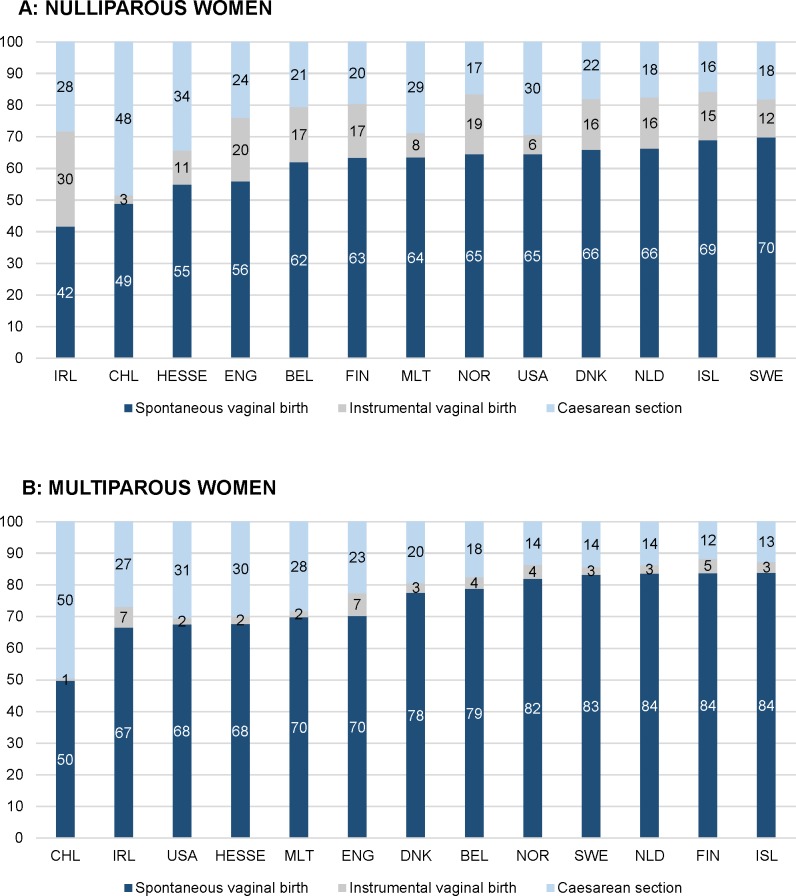
Intercountry variation of mode of birth in 2013 (%). (A) Nulliparous women. (B) Multiparous women. BEL, Belgium; CHL, Chile; DNK, Denmark; ENG, England; FIN, Finland; HESSE, the state of Hesse (Germany); IRL, Ireland; ISL, Iceland; MLT, Malta; NLD, the Netherlands; NOR, Norway; SWE, Sweden; USA, United States of America.

**Fig 3:**
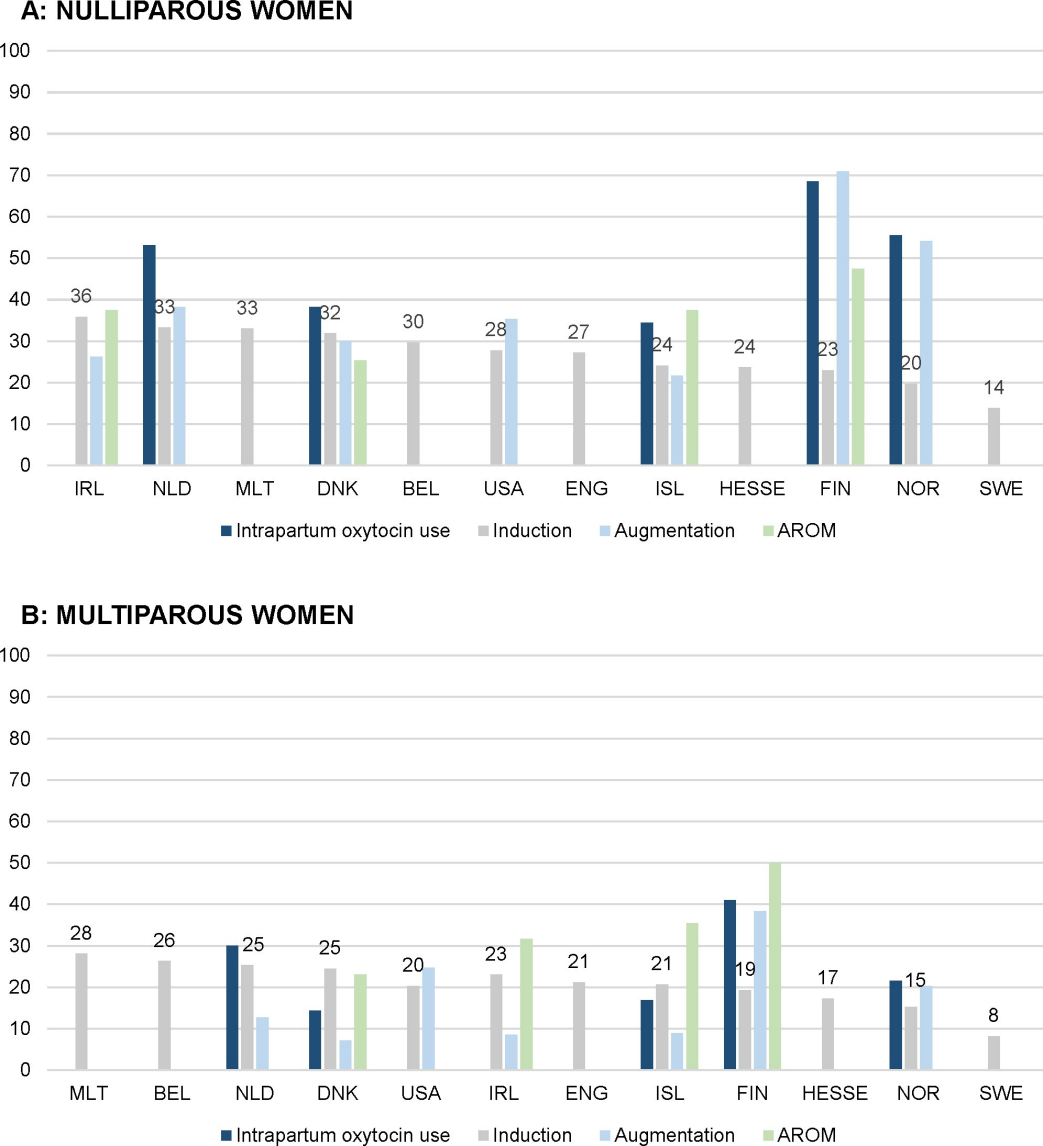
Intercountry variation of interventions to stimulate uterine contractions in 2013 (%). (A) Nulliparous women. (B) Multiparous women. AROM, artificial rupture of membranes; BEL, Belgium; CHL, Chile; DNK, Denmark; ENG, England; FIN, Finland; HESSE, the state of Hesse (Germany); IRL, Ireland; ISL, Iceland; MLT, Malta; NLD, the Netherlands; NOR, Norway; SWE, Sweden; USA, United States of America.

**Fig 4:**
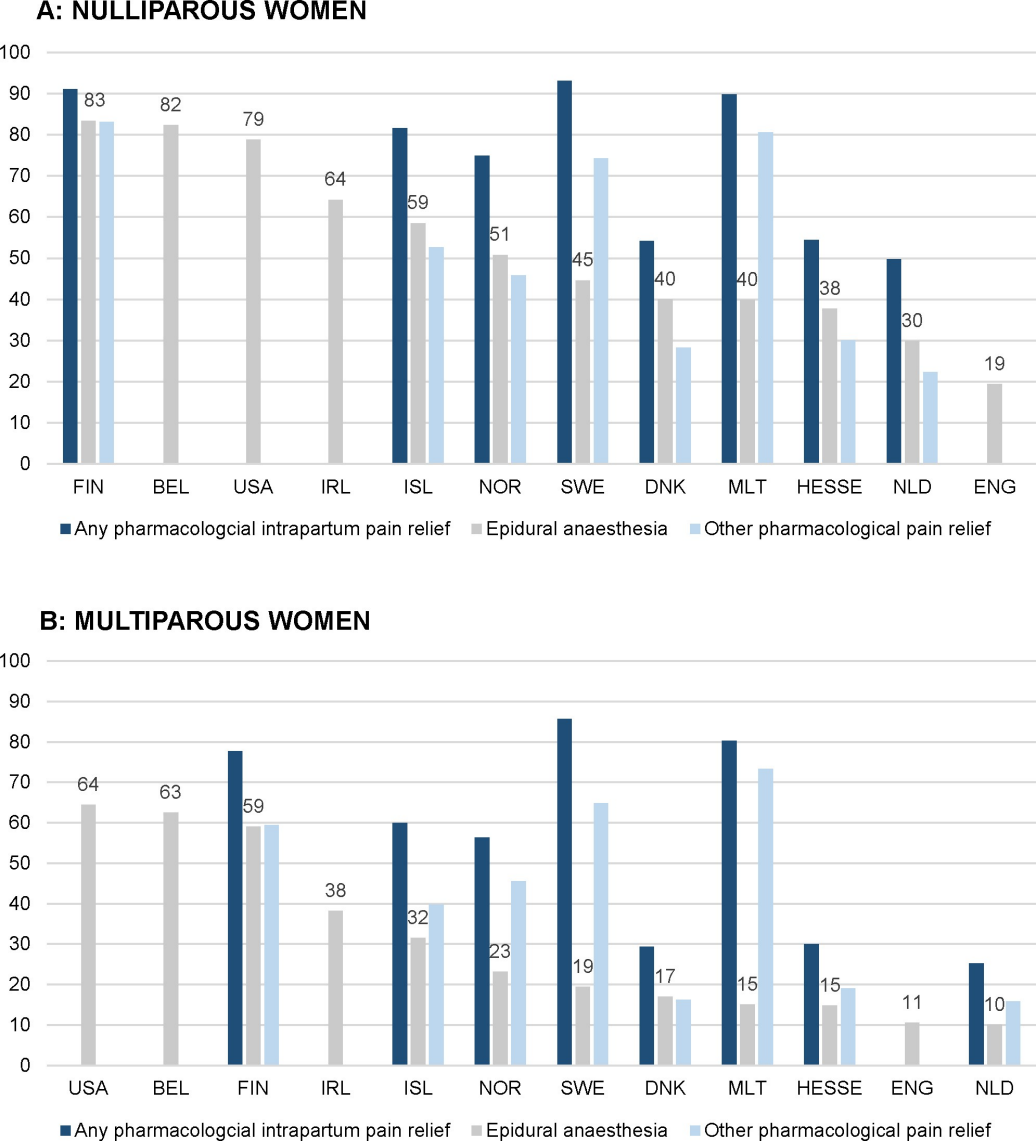
Intercountry variation of pain medication during labour in 2013 (%). (A) Nulliparous women. (B) Multiparous women. BEL, Belgium; CHL, Chile; DNK, Denmark; ENG, England; FIN, Finland; HESSE, the state of Hesse (Germany); IRL, Ireland; ISL, Iceland; MLT, Malta; NLD, the Netherlands; NOR, Norway; SWE, Sweden; USA, United States of America.

**Fig 5:**
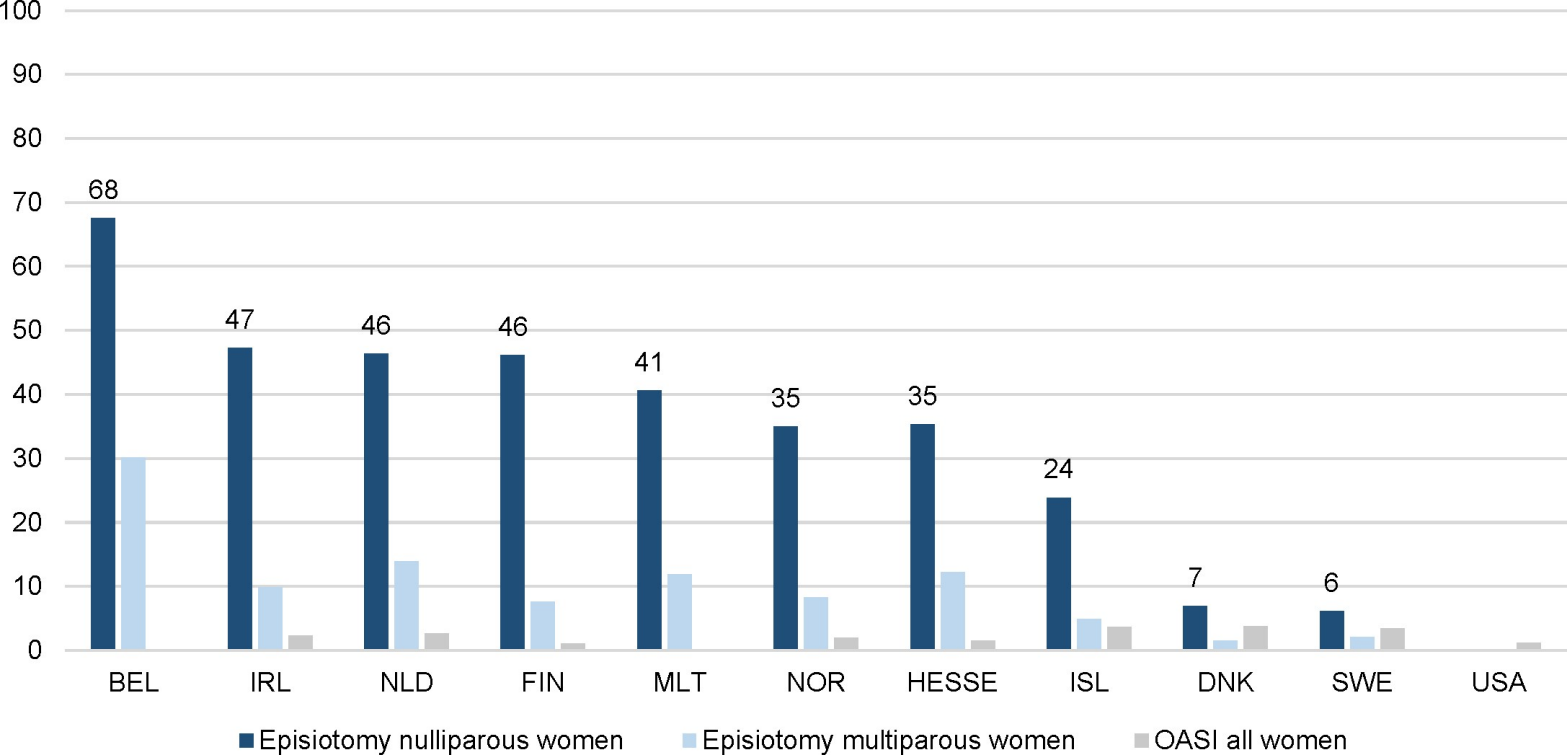
Intercountry variation of episiotomy and OASI in 2013 (%).BEL, Belgium; CHL, Chile; DNK, Denmark; ENG, England; FIN, Finland; HESSE, the state of Hesse (Germany); IRL, Ireland; ISL, Iceland; OASI, obstetric anal sphincter injury; MLT, Malta; NLD, the Netherlands; NOR, Norway; SWE, Sweden; USA, United States of America.

Large variations were found between countries for all interventions. Tables 3 and 4 show intervention rates by country for nulliparous and multiparous women, respectively, and Table 5 shows rates of adverse maternal and neonatal outcomes by country. Figs 1A–5 illustrate incidences in ascending or descending order. Tables 6–8 show Spearman’s rank correlation coefficients. National and data characteristics can be found in S1 Table and unadjusted and adjusted ORs with 99% CIs can be found in S2–S8 Tables. The largest variations, based on the unadjusted ORs, were found for augmentation of labour, pain relief, episiotomy, instrumental vaginal birth, and CS (S2 Table). The ORs varied from 0.50 (99% CI 0.45–0.55) to 3.23 (99% CI 3.14–3.32) for augmentation of labour, from 0.37 (99% CI 0.36–0.37) to 3.86 (99% CI 3.78–3.94) for epidural anaesthesia, and from 0.28 (99% CI 0.27–0.28) to 4.06 (99% CI 3.69–4.47) for other pharmacological pain relief. The ORs varied from 0.19 (99% CI 0.18–0.21) to 4.02 (99% CI 3.91–4.49) for episiotomy, from 0.20 (99% CI 0.19–0.21) to 2.28 (99% CI 2.21–2.36) for instrumental vaginal birth, and from 0.56 (99% CI 0.51–0.63) to 3.34 (99% CI 3.29–3.40) for CS.

**Table 3:** Primary outcomes among nulliparous women: Intervention rates by country in 2013.

All nulliparous women													
Outcomes	FIN	SWE	NOR	DNK	ISL	IRL	ENG	NLD	BEL	HESSE	MLT	USA	CHL
**Total *n***	21,942	48,834	22,870	23,533	1,579	23,722	163,800	68,187	48,722	22,351	1,926	1,393,855	76,324
**Onset of labour**^**$**^**, %**													
**Spontaneous**	**71.8**	**80.1**	**76.4**	**61.0**	**73.1**	**56.9**	**66.1**	**62.9**	**63.3**	**65.1**	**55.8**	**57.4**	-
**Induction of labour by**	**23.0**	**13.8**	**19.7**	**31.9**	**24.1**	**35.9**	**27.2**	**33.3**	**29.7**	**23.7**	**33.0**	**27.8**	-
Cervical ripening	9.1	-	13.6	24.4	20.7	-	-	11.2	-	9.1	-	-	-
AROM	12.3	-	5.4	8.6	2.2	-	-	11.9	-	0.9	-	-	-
Oxytocin	17.6	-	2.4	12.4	12.7	-	-	28.1	-	-	-	-	-
Other method	-	-	7.3	-	-	-	-	-	-	-	-	-	-
Unspecified	-	13.8	-	-	0.1	35.9	27.2	-	29.7	23.6	33.0	27.8	-
Missing, %	0.00	0.00	0.00	0.00	0.00	0.00	2.1	2.8	0.02	0.00	0.00	0.1	
**Prelabour CS**^**#**^	**5.2**	**6.1**	**4.0**	**7.1**	**2.8**	**7.2**	**6.7**	**4.7**	**7.0**	**11.2**	**11.2**	**12.8**	-
Missing onset of labour, %	0.00	0.00	0.01	0.00	0.00	0.00	0.9	2.6	0.04	0.00	0.00	7.0	
Augmentation after spontaneous onset of labour*, **%**	**70.9**	*-*	**54.2** ^	**30.0**	**21.7**	**26.2**	*-*	**38.3**	-	-	-	**35.3**	-
Missing, %	0.00		0.00	0.00	0.00	0.00		1.0				7.0	
**Intrapartum use of oxytocin, %**	**68.5**	*-*	**55.6**	**38.2**	**34.4**	-	*-*	**53.2**	-	-	-	-	-
Missing, %	0.00		0.00	0.00	0.00			1.9					
**Artificial rupture of membranes, %**	**47.5**	*-*	-	**25.4**	**37.5**	**37.5**	*-*	**-**	-	-	-	-	-
Missing, %	0.00			0.00	0.00	0.00		43.9					
**Intrapartum antibiotics, %**	-	*-*	-	-	-	-	-	-	-	-	-	**21.8**	-
Missing, %												9.7	
Any pharmacological intrapartum pain relief*, **%**	**91.1**	**93.1**	**74.9**	**54.2**	**81.6**	-	-	**49.8**	-	**54.4**	**89.8**	-	-
Missing, %	0.00	0.00	0.00	0.00	0.00			2.2		0.00	0.00		
Epidural anaesthesia*, **%**	**83.4**	**44.6**	**50.8**	**40.0**	**58.5**	**64.2**	**19.4**	**29.9**	**82.3**	**37.7**	**39.9**	**78.8**	-
Missing, %	0.00	0.00	0.00	0.00	0.00	0.00	0.9	2.2	0.1	0.00	0.00	9.7	
Other pharmacological pain relief*^$^, **%**	**83.1**	**74.3**	**45.9**	**28.3**	**52.7**	-	-	**22.3**	-	**30.0**	**80.6**	-	-
Systemic (non-) opioid analgesia	-	**-**	0.3	6.3	-	-	-	-	-	-	41.9	-	-
Inhaled nitrous oxide	66.2	74.3	40.6	22.0	52.7	-	-	-	-	-	72.9	-	-
Other	-	-	8.4	-	-	-	-	-	-	-	-	-	-
Unspecified	17.0	-	-	-	-	-	-	22.3	-	30.0	-	-	-
Missing, %	0.00	0.00	0.00	0.00	0.00			2.2		0.00	0.00		
Episiotomy*, **%**	**46.1**	**6.1**	**35.0**	**6.9**	**23.9**	**47.2**	-	**46.4**	**67.5**	**35.3**	**40.6**	-	-
Mediolateral	**-**	-	-	-	-	-	-	46.3	-	31.9	-	-	-
Midline	**-**	-	-	-	-	-	-	0.4	-	3.3	-	-	-
Unspecified	46.1	6.1	35.0	6.9	23.9	47.2	-	-	67.5	0.1	40.6	-	-
Missing, %	0.00	0.00	0.00	0.00	0.00	0.00		1.4	0.1	0.00	0.00		
**Spontaneous vaginal** **birth, %**	**63.4**	**69.9**	**64.6**	**66.0**	**69.0**	**41.7**	**55.9**	**66.3**	**62.0**	**55.0**	**63.5**	**64.6**	**48.9**
Missing, %	0.00	0.00	0.00	0.00	0.00	0.00	0.9	1.0	0.02	0.00	0.00	9.6	0.00
**Instrumental vaginal** **birth, %**	**17.0**	**12.0**	**18.9**	**16.0**	**15.3**	**30.0**	**20.1**	**16.2**	**17.4**	**10.7**	**7.8**	**6.0**	**2.7**
Vacuum extraction	16.2	-	16.4	15.9	15.0	21.2	8.9	-	15.0	9.5	7.1	4.9	0.0
Forceps delivery	0.0	-	2.5	0.1	0.2	8.5	11.2	-	2.4	1.2	0.7	1.1	2.7
Unspecified	0.7	12.0	-	-	0.1	0.4	-	16.2	-	-	-	-	-
Missing, %	0.00	0.00	0.00	0.00	0.00	0.00	0.9	1.0	0.02	0.00	0.00	9.6	0.00
**Cesarean section, %**	**19.6**	**18.1**	**16.5**	**21.5**	**15.8**	**28.3**	**24.0**	**17.5**	**20.5**	**34.3**	**28.8**	**29.5**	**48.4**
Prelabour	5.2	6.1	4.0	7.1	2.8	7.2	6.7	4.6	7.0	11.2	11.2	13.1	-
Emergency during labour	14.4	12.0	12.5	14.3	13.0	21.1	17.3	12.9	13.5	22.9	17.6	15.8	-
Unspecified	-	-	-	-	-	-	-	-	0.0	4.2	-	0.5	48.4
Missing, %	0.00	0.00	0.00	0.00	0.00	0.00	0.9	1.0	0.02	0.00	0.00	9.6	0.00
**Active management of third stage of labour, %**	-	-	-	-	-	-	-	-	-	-	-	-	-
**Use of oxytocin postpartum, %**	-	-	-	-	-	-	-	*-*	-	-	-	-	-
Missing, %								43.3					

Main interventions are indicated in bold type.

*Place of birth:

• FIN: other place of birth: on the way to the hospital, unplanned births outside the hospital.

• NOR: ‘hospital’ includes small birth centres; other place of birth: during transport or unspecified place outside the institution (baby born before arrival).

• BEL: other place of birth: in a car, ambulance, on the way to the hospital.

• MLT: other place of birth: in a car, ambulance, on the way to the hospital, sometimes on a boat or at an emergency department.

• ENG: HIPE records hospital births only.

^$^HESSE: based on a total of 45,393 births. Only the births in the hospital or ‘other’ place are included in this study (*n* = 44,722), because a separate database with out-of-hospital births could not be linked.

^#^Care provider:

• NOR: at onset of labour: midwives are the responsible care provider in most of births without prelabour CS; at birth: midwives are the responsible care provider in most of spontaneous vaginal births (these numbers are based on the variables prelabour CS and spontaneous vaginal births).

• USA: other care provider: 6.2% of births are under responsibility of a doctor of osteopathy.

• CHL: in the data of Chile, it was only registered which care provider attended the birth. Although obstetricians are the last responsible care providers, midwives attend all normal births.

^CHIL: birth is defined as birth from 24 weeks onwards, instead of 16 weeks.

Abbreviations: AROM, artificial rupture of membranes; BEL, Belgium; CHL, Chile; DNK, Denmark; ENG, England; FIN, Finland; HESSE, the state of Hesse (Germany); IRL, Ireland; ISL, Iceland; MLT, Malta; NLD, the Netherlands; NOR, Norway; SWE, Sweden; USA, United States of America

**Table 4:** Primary outcomes among multiparous women: intervention rates by country in 2013.

All multiparous women													
Outcomes	FIN	SWE	NOR	DNK	ISL	IRL	ENG	NLD	BEL	HESSE	MLT	USA	CHL
**Total *n***	32,368	55,736	32,081	27,159	2,408	38,870	246,566	84,457	64,185	22,371	1,855	2,106,542	97,153
**Onset of labour**^**$**^**, %**													
**Spontaneous**	**74.1**	**82.0**	**77.0**	**61.3**	**71.3**	**57.3**	**65.5**	**65.7**	**60.8**	**66.3**	**50.9**	**52.3**	-
**Induction of labour by**	**19.3**	**8.2**	**15.2**	**24.5**	**20.7**	**23.0**	**21.2**	**25.3**	**26.3**	**17.3**	**28.1**	**20.3**	-
Cervical ripening	5.4	-	9.5	16.1	13.9	-	-	6.6	-	6.1	-	-	-
AROM	12.2	-	5.6	10.3	5.7	-	-	14.0	-	1.2	-	-	-
Oxytocin	12.6	-	1.3	7.4	8.0	-	-	20.7	-	-	-	-	-
Other method	-	-	4.6	-	-	-	-	-	-	-	-	-	-
Unspecified	-	8.2	-	-	0.1	23.0	21.2	-	26.3	16.9	-	20.3	-
Missing, %	0	0.0	0.0	0.0	0.0	0.0	1.1	1.8	0.01	0.0	0.0	0.1	
**Prelabour CS**^**#**^	**6.5**	**9.7**	**7.8**	**14.2**	**8**	**19.7**	**13.4**	**9**	**12.8**	**16.4**	**21**	**25.7**	-
Missing onset of labour, %	0.0	0.0	0.0	1.1	0.0	0.0	1.0	1.7	0.1	0.0	0.0	7.9	
Augmentation after spontaneous onset of labour*, *%*	**38.3**	-	**20.3**^	**7.2**	**8.9**	**8.5**	-	**12.7**	-	-	-	**24.7**	-
Missing, %	0.0		0.0	0.0	0.0	0.0		1.3				7.9	
**Intrapartum use of oxytocin, *%***	**41**	-	**21.6**	**14.4**	**16.9**	-	-	**30**	-	-	-	-	-
Missing, %	0.0		0.0	0.0	0.0			2.1					
**Artificial rupture of membranes, *%***	**49.9**	-	-	**23**	**35.4**	**31.7**	-	**-**	-	-	-	-	-
Missing, %	0.0			0.0	0.0	0.0		47.9					
**Intrapartum antibiotics, %**	-	-	-	-	-	-	-	-	-	-	-	**19.2**	-
Missing, %												9.7	
Any pharmacological intrapartum pain relief*, **%**	**77.7**	**85.7**	**56.3**	**29.4**	**60**	-	-	**25.2**	-	**30**	**80.3**	-	-
Missing, %	0.0	0.0	0.0	0.0	0.0			1.7		0.0	0.0		
Epidural anaesthesia*, **%**	**59.0**	**19.4**	**23.2**	**17.0**	**31.5**	**38.2**	**10.6**	**10.0**	**62.5**	**14.8**	**15.1**	**64.4**	-
Missing, %	0	0	0	0	0	0	1	1.7	0.2	0	0.1	9.7	
Other pharmacological pain relief*^$^, **%**	**59.4**	**64.9**	**45.5**	**16.2**	**39.7**	-	-	**15.8**	-	**19.1**	**73.3**	-	-
Systemic (non-)opioid analgesia	-	-	0.2	1.5	-	-	-	-	-	-	33.1	-	-
Inhaled nitrous oxide	51.3	64.9	34.9	14.7	39.7	-	-	-	-	-	65.1	-	-
Other	-	-	10.9	-	-	-	-	-	-	-	-	-	-
Unspecified	8.1	-	-	-	-	-	-	15.8	-	19.1	-	-	-
Missing, %	0.0	0.0	0.0		0.0			1.7		0.0	0.1		
Episiotomy*, **%**	**7.6**	**2.1**	**8.3**	**1.5**	**4.9**	**9.9**	-	**13.9**	**30.2**	**12.2**	**11.9**	-	-
Mediolateral	-	-	-	-	-	-	-	13.7	-	10.5	-	-	-
Midline	-	-	-	-	-	-	-	0.1	-	1.6	-	-	-
Unspecified	7.6	2.1	8.3	1.5	4.9	9.9	-	-	30.2	0.03	11.9	-	-
Missing, %	0.0	0.0	0.0	0.0	0.0	0.0		2.0	0.1	0.0	0.0		
**Spontaneous vaginal** **birth, %**	**83.8**	**83.2**	**82.0**	**77.6**	**83.9**	**66.6**	**70.2**	**83.6**	**78.8**	**67.7**	**69.9**	**67.6**	**49.8**
Missing, %	0	0.0	0.0	0.0	0.0	0.0	1.0	0.5	0.03	0.0	0.0	9.6	0.0
**Instrumental vaginal** **birth, %**	**4.5**	**2.5**	**4.4**	**3.0**	**3.4**	**6.5**	**7.2**	**2.7**	**3.7**	**2.4**	**1.9**	**1.9**	**0.7**
Vacuum extraction	4.0	-	3.8	3.0	3.3	5.4	3.4	-	3.2	2.2	1.7	1.6	0.0
Forceps delivery	0.01	-	0.6	0.1	0.1	1.1	3.8	-	0.5	0.2	0.2	0.3	0.7
Unspecified	0.5	2.5	-	-	-	0.04	-	2.7	-	-	-	-	-
Missing, %	0.0	0.0	0.0	0.0	0.0	0.0	1.0	0.5	0.03	0.0	0.0	9.6	0.0
**CS, %**	**11.7**	**14.3**	**13.6**	**19.9**	**12.8**	**26.9**	**22.6**	**13.6**	**17.7**	**29.9**	**28.2**	**30.5**	**49.5**
Prelabour	6.5	9.7	7.8	14.2	8.0	19.7	13.2	8.9	12.8	16.4	21.0	26.2	-
Emergency during labour	5.2	4.6	5.8	5.8	4.8	7.2	9.3	4.8	4.8	9.3	7.2	3.7	-
Unspecified	-	-	-	-	-	-	-		0.02	0.2	-	0.5	49.5
Missing, %	0.0	0.0	0.0	0.0	0.0	0.0	1.0	0.5	0.03	0.0	0.0	9.6	0.0
**Active management of third stage of labour, %**	-	-	-	-	-	-	-	-	-	-	-	-	-
**Use of oxytocin postpartum, %**	-	-	-	-	-	-	-	-	-	-	-	-	-
Missing, %								47.6					

Main interventions are indicated in bold type.

^#^Incidences of prelabour CS as a part of ‘onset of labour’ can be slightly different to prelabour CS as a part of ‘cesarean section’, because of different denominators due to missing data.

*Denominators:

• Augmentation of labour: women with a spontaneous onset of labour (planned CS and induction of labour are excluded from the denominator).

• Pain relief (any pharmacological pain relief, epidural, and other pain relief): women with an attempted vaginal birth (planned CS is excluded from the denominator).

• Episiotomy: women with a vaginal birth (CS is excluded from the denominator).

^$^Induction of labour and other pharmacological pain relief: total percentage can exceed 100, because more than one method can be used.

^NOR: augmentation of labour was based on a proxy variable; there is a certain amount of uncertainty.

Abbreviations: AROM, artificial rupture of membranes; BEL, Belgium; CHL, Chile; CS, cesarean section; DNK, Denmark; ENG, England; FIN, Finland; HESSE, the state of Hesse (Germany); IRL, Ireland; ISL, Iceland; MLT, Malta; NLD, the Netherlands; NOR, Norway; SWE, Sweden; USA, United States of America

**Table 5:** Adverse maternal and neonatal outcomes by country in 2013.

Outcomes	FIN	SWE	NOR	DNK	ISL	IRL	ENG	NLD	BEL	HESSE	MLT	USA	CHL
Total	54,310	104,570	54,951	50,692	3,987	62,613	410,366	152,644	112,907	44,722	3,781	3,500,397	173,477
**Perinatal mortality up to 7 days, %**	**0.13**	**0.13**	**0.14**	**0.16**	**0.08**	Ante- and intrapartum mortality: **0.39**	-	**0.17**	**0.16**	Ante- and intrapartum mortality^$^: **0.16**	**0.21**	-	-
Missing, %	0.0	0.2	0.0	0.0	0.0	0.0		0.01	0.0	0.0	0.0		
Apgar score below 7 at 5 minutes*, %	**1.68**	**1.33**	**0.93**	**0.56**^**#**^	**1.78**	-	-	**1.08**	**1.18**	**0.64**	**0.58**	**1.19**	**0.75**
Missing, %	0.2	2.1	0.02	0.7	0.0			<0.01	0.02	0.0	0.1	0.4	1.8
**Maternal mortality up to 7 days, %**	**2/100,000**	**3/100,000**	-	**0/100,000**	**0/100,000**	-	-	**1/100,000**	-	**0/100,000**	**0/100,000**	-	Mortality until 42 days: **20/100,000**
Missing, %	0.0	0.0		0.0	0.0			0.0	0.0	0.0	0.0		
**Postpartum haemorrhage ≥ 1,000 ml, %**	**-**	**-**	-	-	**4.96**	-	-	**5.96**	-	**1.20**	**1.08**	-	-
Missing, %					12.5			3.2		0.0	0.0		
**Obstetric anal sphincter injury, %**	**1.12**	**3.47**	**1.96**	**3.73**	**3.70**	**2.30**	-	**2.71**	-	**1.48**	-	**1.24**	-
Missing, %	0.0	0.0	0.0	0.0	0.0	0.0		2.2		0.0		9.7	

* Denominators:

• Apgar score below 7 at 5 minutes: antepartum mortality was excluded, or an Apgar score of zero was excluded if antepartum mortality was not registered (this was the case for DNK, NOR, and USA).

• Obstetric anal sphincter injury: women with a vaginal birth (CS is excluded from the denominator).

^$^HESSE: mortality is not registered after a woman is discharged from the hospital: the incidence is of antepartum, intrapartum, and neonatal mortality until discharge at mostly approximately 2 days postpartum.

^#^DNK: Apgar score at 5 minutes was not always registered, especially in cases with low Apgar score; the percentage may therefore be higher.

Abbreviations: BEL, Belgium; CHL, Chile; CS, cesarean section; DNK, Denmark; ENG, England; FIN, Finland; HESSE, the state of Hesse (Germany); IRL, Ireland; ISL, Iceland; MLT, Malta; NLD, the Netherlands; NOR, Norway; SWE, Sweden; USA, United States of America

**Table 6:** Correlations between interventions among nulliparous women tested with Spearman’s rho (two-tailed significance level).

	Hospital birth (all births)	Spontaneous onset of labour	Induction of labour	Prelabour CS	Epidural anaesthesia*	Other pain relief*	Episiotomy in vaginal births	Spontaneous vaginal birth	Instrumental vaginal birth	CS	Emergency CS during labour
	**Nulliparous women**										
**Births at ≥42 weeks** (all births)	rho = −0.43	**rho = 0.82**	**rho = −0.71**	**rho = −0.61**	rho = −0.04	rho = 0.38	rho = −0.55	rho = 0.44	rho = 0.56	**rho = −0.61**	rho = −0.46
**Spontaneous onset of labour**	rho = −0.22										
**Induction of labour**	rho = −0.03	**rho = −0.88**									
**Prelabour CS**	rho = 0.24	**rho = −0.73**	rho = 0.39								
Epidural anaesthesia*	**rho = 0.62**	rho = 0.05	rho = −0.14	rho = −0.02							
Other pain relief*	**rho = 0.62**	rho = 0.24	rho = −0.43	rho = 0.07	**rho = 0.64**						
**Episiotomy in vaginal births**	rho = 0.33	rho = −0.50	rho = 0.56	rho = 0.19	rho = 0.28	rho < 0.01					
**Spontaneous vaginal birth**	rho = −0.43	rho = 0.37	rho = −0.26	rho = −0.49	rho = −0.06	rho = −0.17	**rho = −0.68**				
**Instrumental vaginal birth**	rho = −0.29	rho = 0.17	rho = 0.13	rho = −0.42	rho = 0.08	rho = −0.17	rho = 0.50	rho = −0.17			
**CS**	rho = 0.34	**rho = −0.66**	rho = 0.32	**rho = 0.96**	rho = −0.13	rho = 0.07	rho = 0.26	**rho = −0.70**	rho = −0.47		
**Emergency CS during labour**	rho = 0.16	**rho = −0.62**	rho = 0.39	**rho = 0.74**	rho = −0.11	rho = 0.14	rho = 0.39	**rho = −0.81**	rho = −0.07	**rho = 0.87**	

All correlations are based on crude ORs of the intervention rates of the countries. The correlations are therefore on country level and not on an individual-woman level.

Correlations are only measured between interventions of which eight or more countries were included and if ORs were significantly different.

Correlations with rho ≥ 0.60 or ≤ −0.60 are indicated in bold type, since they are considered strong.

*Measured in a group of women without prelabour CS.

Abbreviations: CS, cesarean section; OR, odds ratio

**Table 7:** Correlations between interventions among multiparous women tested with Spearman’s rho (two-tailed significance level).

	Hospital birth (all births)	Spontaneous onset of labour	Induction of labour	Prelabour CS	Epidural anaesthesia*	Other pain relief*	Episiotomy in vaginal births	Spontaneous vaginal birth	Instrumental vaginal birth	CS	Emergency CS during labour
	**Multiparous women**										
**Births at ≥42 weeks** (all births)		**rho = 0.83**	**rho = −0.66**	**rho = −0.65**	rho = −0.05	rho = 0.26	**rho = −0.67**	**rho = 0.66**	**rho = 0.67**	**rho = −0.67**	rho = −0.04
**Spontaneous onset of labour**	rho = −0.18										
**Induction of labour**	rho = 0.11	**rho = −0.76**									
**Prelabour CS**	rho = −0.04	**rho = −0.82**	rho = 0.35								
Epidural anaesthesia*	rho = 0.52	rho = −0.13	rho = −0.14	rho < 0.01							
Other pain relief*	rho = 0.14	rho = 0.29	rho = −0.21	rho = 0.05	rho = 0.43						
**Episiotomy in vaginal births**	rho = 0.08	rho = −0.47	rho = 0.48	rho = 0.26	rho = −0.09	rho = −0.19					
**Spontaneous vaginal birth**	rho = −0.29	**rho = 0.70**	rho = −0.17	**rho = −0.90**	rho = −0.04	rho = −0.05	rho = −0.28				
**Instrumental vaginal birth**	rho = −0.25	rho = 0.23	rho = −0.03	rho = −0.47	rho = 0.11	rho = −0.14	rho = −0.10	rho = 0.36			
**CS**	rho = 0.18	**rho = −0.70**	rho = 0.18	**rho = 0.96**	rho = −0.06	rho < 0.01	rho = 0.30	**rho = −0.95**	rho = −0.56		
**Emergency CS during labour**	rho = 0.05	rho = −0.16	rho = 0.14	rho = 0.22	rho = −0.51	rho < 0.01	rho = 0.19	rho = −0.43	rho = 0.33	rho = 0.32	

All correlations are based on crude ORs of the intervention rates of the countries. The correlations are therefore on country level and not on an individual-woman level.

Correlations are only measured between interventions of which eight or more countries were included and if ORs were significantly different.

Correlations with rho ≥ 0.60 or ≤ −0.60 are indicated in bold type, since they are considered strong.

*Measured in a group of women without prelabour CS.

Abbreviations: CS, cesarean section; OR, odds ratio

**Table 8:** Correlations between interventions and adverse outcomes tested with Spearman’s rho (two-tailed significance level).

	**Apgar score below 7 at 5 minutes**	**Obstetric anal sphincter injury in vaginal births**		
**All women**				
**Births at ≥42 weeks**	rho = 0.51	rho = 0.17		
**Hospital birth**	rho = −0.27	rho = −0.45		
	**Apgar score below 7 at 5 minutes**	**Obstetric anal sphincter injury in vaginal births**	**Apgar score below 7 at 5 minutes**	**Obstetric anal sphincter injury in vaginal births**
	**Nulliparous women**		**Multiparous women**	
**Spontaneous onset of labour**	rho = 0.53	rho = 0.12	rho = −0.44	rho = 0.05
**Induction of labour**	rho = −0.45	rho = 0.22	rho = −0.31	rho = 0.37
Epidural anaesthesia*	**rho = 0.64**	rho = −0.50	rho = 0.59	rho = −0.48
**Other pharmacological pain relief**	rho = 0.38	rho = −0.32	rho = 0.14	rho = −0.29
**Episiotomy in vaginal births**	rho = 0.07	rho = −0.57	rho = −0.07	rho = −0.57
**Spontaneous vaginal birth**	rho = 0.37	**rho = 0.65**	**rho = 0.64**	rho = 0.22
**Instrumental vaginal birth**	rho = 0.21	rho = 0.02	rho = 0.40	rho = 0.03
**CS**	rho = −0.55	rho = −0.43	rho = −0.59	rho = −0.12

All correlations are based on crude ORs of the intervention rates of the countries. The correlations are therefore on country level and not on an individual-woman level.

Correlations are only measured between interventions of which eight or more countries were included and if ORs were significantly different.

Correlations with rho ≥ 0.60 or ≤ −0.60 are indicated in bold type, since they are considered strong.

*Measured in a group of women without prelabour CS.

Abbreviations: CS, cesarean section; OR, odds ratio

#### Onset of labour and mode of birth

**Fig 6:**
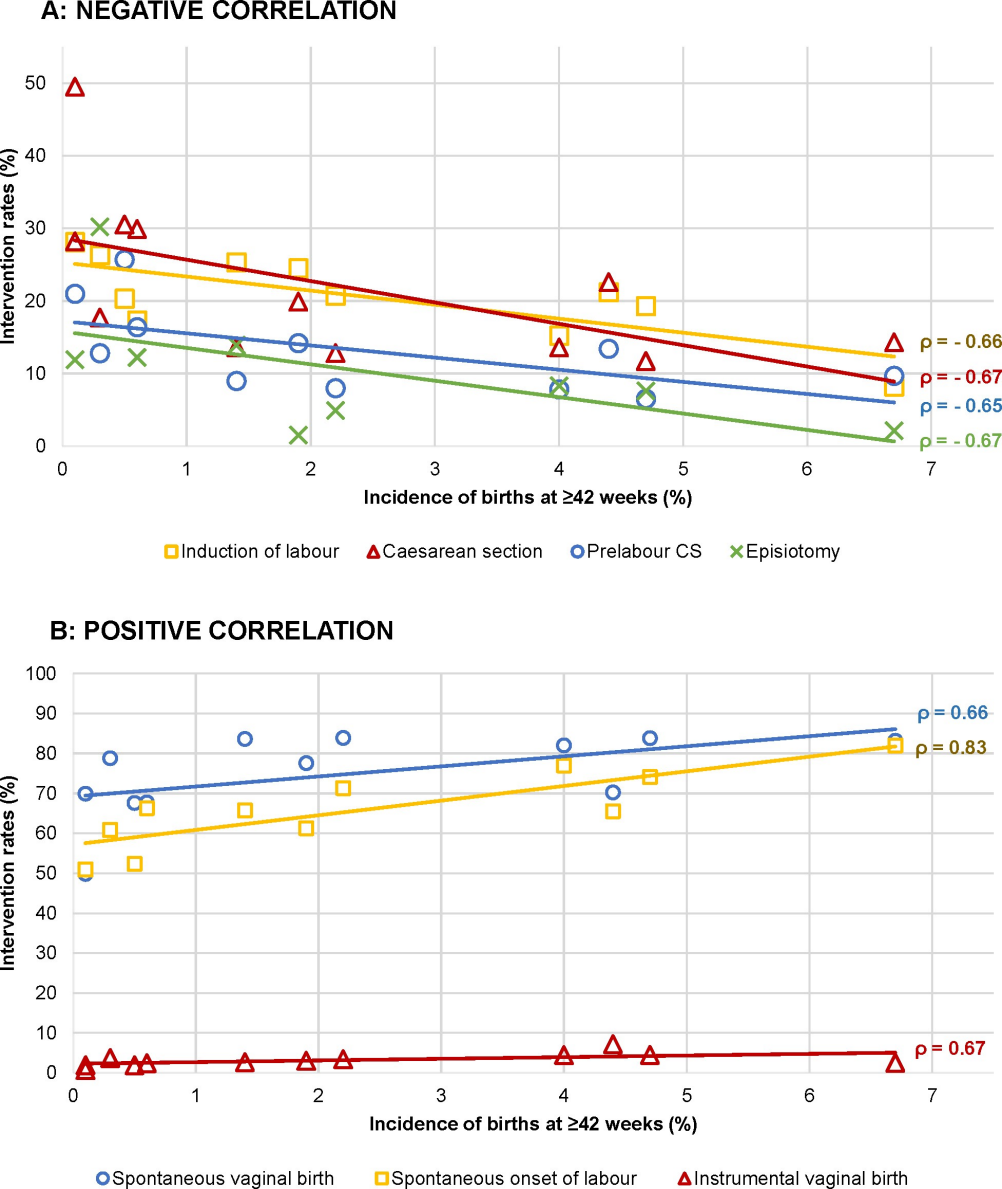
Interventions among multiparous women with a correlation with the incidence of births at ≥42 weeks’ gestation. (A) Negative correlation. (B) Positive correlation. CS, cesarean section.

**Fig 7:**
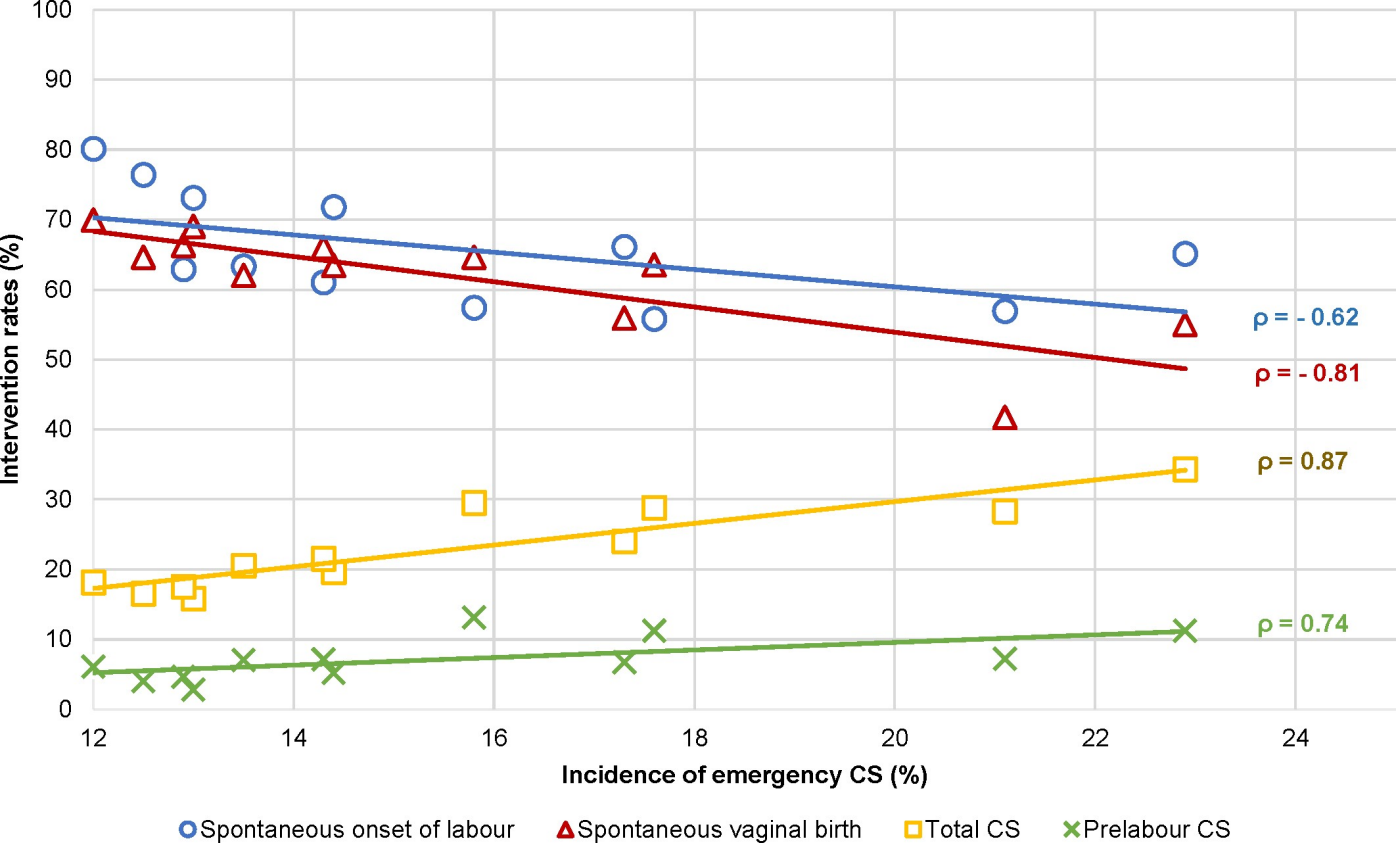
Interventions among nulliparous women with a correlation with emergency CS. CS, cesarean section.

A higher incidence of births at ≥42 weeks’ gestation correlated with higher rates of births with a spontaneous onset of labour (rho = 0.82 for nulliparous and rho = 0.83 for multiparous women) and instrumental (rho = 0.70) and spontaneous (rho = 0.66) vaginal births among multiparous women and with lower rates of induction of labour (rho = −0.14 and rho = −0.66), prelabour CS (rho = −0.61 and rho = −0.65), episiotomy among multiparous women (rho = −0.67), and overall CS (rho = −0.61 and rho = −0.67) (Tables 6 and 7 and Fig 6A and 6B). Rates of births with a spontaneous onset varied from 55.8% to 80.1% among nulliparous women and from 50.9% to 82.0% among multiparous women in Malta and Sweden, respectively (Tables 3 and 4 and Fig 1A and 1B). Countries with higher rates of births with a spontaneous onset had lower rates of overall CS (rho = −0.66 for nulliparous [Table 6] and rho = −0.70 for multiparous [Table 7] women), lower rates of emergency CS among nulliparous women (rho = −0.62), and higher rates of spontaneous vaginal births among multiparous women (rho = 0.70). Variation in spontaneous onset of labour was due to variation in both induction of labour and prelabour CS. Rates of induction of labour among nulliparous women varied from 13.8% in Sweden to 35.9% in Ireland and from 8.2% in Sweden to 28.1% in Malta among multiparous women, with considerable variation in the methods used for induction. Rates of prelabour CS among nulliparous women varied across countries, from 2.8% in Iceland to 12.8% in the USA and from 6.5% in Finland to 25.7% in the USA among multiparous women (Fig 1A and 1B). Correlations between prelabour and overall CS rates were rho = 0.96 for both nulliparous and multiparous women, and between emergency and overall CS the correlations were rho = 0.74 and rho = 0.22, respectively (Tables 6 and 7). Strong correlations between CS and other variables are illustrated in Fig 7. Percentages of emergency CS performed after the onset of labour varied between 12.0% in Sweden and 22.9% in the state of Hesse (Germany) among nulliparous women and between 3.7% in the USA and 9.3% in England and the state of Hesse (Germany) among multiparous women (Fig 2A and 2B).

Percentages of spontaneous vaginal births varied between 41.7% in Ireland to 69.9% in Sweden among nulliparous women and between 49.8% in Chile to 83.9% in Iceland among multiparous women (Fig 2A and 2B). Percentages of instrumental vaginal births varied from 2.7% in Chile to 30.0% in Ireland among nulliparous women and from 0.7% in Chile to 7.2% in England among multiparous women. A positive correlation was found between prelabour CS and emergency CS rates among nulliparous women (rho = 0.74), and a negative correlation was found between prelabour CS and spontaneous vaginal birth among multiparous women (rho = −0.90) (Tables 6 and 7).

#### Other interventions

Augmentation of labour was recorded in seven of 13 countries and varied from 21.7% in Iceland to 70.9% in Finland among nulliparous women and from 7.2% in Denmark to 38.3% in Finland among multiparous women. Among nulliparous women, oxytocin for induction or augmentation of labour rates ranged from 34.4% in Iceland to 68.5% in Finland and, among multiparous women, ranged from 14.4% in Denmark to 41.0% in Finland. Rates of artificial rupture of membranes varied from 25.4% to 47.5% among nulliparous women and from 23.0% to 49.9% among multiparous women in Denmark and Finland, respectively, but this variable was recorded in only five countries, and one of those had more than 10% missing data (Tables 3 and 4 and Fig 3A and 3B).

Fig 4A and 4B illustrate the variation in use of intrapartum pharmacological pain relief among nulliparous and multiparous women. Percentages of births with the use of any pharmacological intrapartum pain relief varied from 49.8% to 93.1% among nulliparous women and from 25.2% to 85.7% among multiparous women in the Netherlands and Sweden, respectively. Among nulliparous women, epidural anaesthesia use varied from 19.4% in England to 83.4% in Finland and rates of other pharmacological pain relief varied from 22.3% in the Netherlands to 83.1% in Finland. Among multiparous women, these percentages varied from 10.0% in the Netherlands to 64.4% in the USA and from 15.8% in the Netherlands to 73.3% in Malta (Tables 3 and 4). Among nulliparous women, countries with higher use of epidural anaesthesia use also had higher rates of other pharmacological pain relief among nulliparous women (rho = 0.64), and countries with higher hospital birth rates had higher rates of epidural anaesthesia and other pharmacological pain relief (rho = 0.62) (S1 Fig and S2 Fig).

Use of episiotomy varied from 6.1% in Sweden to 67.5% in Belgium among nulliparous women and from 1.5% in Denmark to 30.2% in Belgium among multiparous women (Fig 5). In countries with higher rates of spontaneous vaginal births, lower rates of episiotomy in vaginal births were found among nulliparous women (rho = −0.68) (Table 6 and S3 Fig).

### Variations in adverse outcomes

Variations in rates of perinatal mortality up to 7 days after birth ranged from 0.08% in Iceland to 0.21% in Malta for all births (Table 5), but this variation was not statistically significant (S2 Table), and the incidence of Apgar score below 7 at 5 minutes varied from 0.58% in Malta to 1.78% in Iceland. The state of Hesse (Germany) and Ireland could only provide antepartum fetal mortality rates (0.16% and 0.39% respectively), and in three countries, data on perinatal mortality rates were missing. The maternal mortality rate up to 7 days postpartum varied between 0/100,000 and 3/100,000. Chile provided the mortality rate until 42 days postpartum, which was 20/100,000. PPH was only recorded in four countries and varied from 1.08% in Malta to 5.96% in the Netherlands. Percentages of OASI in vaginal births varied from 1.12% in Finland to 3.73% in Denmark.

Spearman’s rank correlation coefficients were calculated only for Apgar score < 7 at 5 minutes and OASI, because eight or more countries provided data on these variables (Table 8), and variations for these variables were statistically significant (S3 Table). Percentages of OASI in vaginal births were higher in countries with higher rates of spontaneous vaginal birth among nulliparous women (rho = 0.65) (S3 Fig). In countries with higher rates of epidural anaesthesia among nulliparous women and spontaneous vaginal births among multiparous women, higher rates of Apgar score < 7 were found (rho = 0.64 for both variables) (S4 Fig).

### Adjustments for population characteristics

Multivariable logistic regression analyses showed the magnitude of variations. Data from all countries could be included in the univariable logistic regressions (S2 Table). Data from eight countries could be included in all multivariable analyses, which were Norway, Iceland, Ireland, the Netherlands, Belgium, Malta, USA, and Chile. Comparisons between unadjusted and adjusted ORs did not result in differences in ranking of the countries from highest to lowest rates in most interventions and outcomes. The OR for augmentation increased from 1.62 (99% CI 1.56–1.68) to aOR 1.99 (99% CI 1.92–2.06) for Norway after adjustments for parity, maternal age, and ethnicity, and slightly for the USA, and decreased from OR 0.97 (99% CI 0.94–1.00) to aOR 0.58 (99% CI 0.57–0.61) for the Netherlands (S5 Table). Variation for episiotomy in vaginal births increased after adjustments for parity, maternal age, and ethnicity, mainly for the Netherlands, which increased from OR 1.18 (99% CI 1.14–1.23) to aOR 1.38 (99% CI 1.33–1.43), and for Belgium, which increased from OR 2.57 (99% CI 2.48–2.67) to aOR 3.39 (99% CI 3.27–3.53) (S5 Table). The OR also increased for Belgium after adjustments for parity, maternal age, and BMI, from OR 2.68 (99% CI 2.56–2.81) to aOR 3.65 (99% CI 3.49–3.83) (S6 Table). The OR of OASI decreased for the Netherlands after adjustments, mainly after adjustments for parity, maternal age, and ethnicity, from OR 1.22 (99% CI 1.14–1.31) to aOR 1.05 (99% CI 0.98–1.13) (S3 Table).

### Missing data

CS and instrumental vaginal birth were the only variables recorded in all countries. In many countries, data on several variables were missing (Tables 3 and 4). Administration of antibiotics during labour was only recorded in the USA. Active management of the third stage of labour was not recorded in any of the countries, and artificial rupture of membranes and use of oxytocin postpartum were only recorded in the Netherlands, with more than 10% of the data missing. For all other dependent variables, percentages of missing data were less than 10%.

### Quality of the data

Quality of the data, methods of data collection, and definition of variables varied across countries. In all countries, some data quality assurance procedures were routinely performed, although methods differed across countries. In most countries, a representative sample of the population was provided. Some countries could not provide data for home births, although these were very few in number. In eight countries (Finland, Sweden, Norway, Iceland, Ireland, the state of Hesse [Germany], Malta, and Chile), no distinction could be made between the absence of the outcome and missing cases, because only the presence of the outcome was recorded, whereas both the absence and the missing cases were recorded as an empty field. A certain amount of missing data is expected, which would result in a lower denominator and therefore a higher incidence of that variable. In some cases, data from countries could not be included for some variables because definitions of variables differed substantially. For instance, postpartum blood loss was categorised in different ways, not as ≥1,000 ml, and any perineal injury was recorded as perineal tear but not subdefined as third and fourth degree perineal tears.

## Discussion

In this multinational cross-sectional study, considerable intercountry variation was found for all investigated interventions, even after adjustments for parity, maternal age, ethnicity, maternal BMI, education, and birth weight. The largest variations were found for augmentation of labour, pain relief, episiotomy, instrumental vaginal birth, and CS. Countries with a higher rate of births at ≥42 weeks’ gestation had higher rates of births with a spontaneous onset, spontaneous vaginal births among multiparous women, and instrumental vaginal births and had lower rates of induction of labour, prelabour CS, episiotomy among multiparous women, and overall CS. Lower rates of induction of labour, prelabour CS, overall CS, and emergency CS (nulliparous women only) and higher rates of spontaneous vaginal births among multiparous women were found in countries with higher rates of births with a spontaneous onset. In countries with lower rates of out-of-hospital births, the rates of pain medication were lower among nulliparous women. Variation in the overall CS rate was strongly correlated with variation in prelabour CS, and a positive correlation was found between prelabour CS and emergency CS among nulliparous women. We found higher rates of OASI in vaginal births in countries with higher rates of spontaneous vaginal birth among nulliparous women. Higher rates of Apgar score < 7 were found in countries with higher rates of epidural anaesthesia among nulliparous women and higher rates of spontaneous vaginal births among multiparous women. There was no significant difference in perinatal mortality rates up to 7 days.

### Strengths and limitations

This study is comparable to previous studies on variations in childbirth interventions, such as the EURO-PERISTAT reports [17,18], but included more recent data and data at the individual level, used uniform protocols for data analyses, adjusted for confounders, and investigated correlations between outcomes. Although the 13 countries in our study are not a representative sample of all high-income countries, this study is the first large multinational study that made adjustments for population characteristics and focused on correlations between birth characteristics, interventions, and adverse outcomes. Collecting data at the individual level is still very challenging for many countries. A strength of this study is the inclusion of national data, or a representative sample of national data, on full-term singleton births and transparency about missing data. A uniform protocol for data analysis was used and data from most countries were analysed by the same person or in close contact with the principal investigator, increasing comparability of variables between countries. Furthermore, results were double-checked by both the principal investigator and the investigator from the country.

A limitation of this study is that it used routinely collected data, and consequently, the quality of the data varied across countries. Despite extensive recommendations of EURO-PERISTAT [17,18] and the World Health Organization (WHO) [19] on maternal and perinatal indicators and definitions, routinely collected data still vary substantially. In many countries, several recommended indicators, including those that are defined as core indicator by EURO-PERISTAT [17–20], are missing from routinely collected data sets, and coding of variables such as induction and augmentation of labour and type of CS are inconsistently recorded across countries. Data on maternal characteristics such as ethnicity, BMI, education, and socioeconomic status are missing in several countries, resulting in limitations in adjusting for these potential confounders. Potential maternal confounders other than those in our study, such as smoking, preexisting medical conditions and previous obstetric history, and indications for the use of interventions, are missing in most routinely collected data sets. Comparisons of indications of interventions are important in order to investigate which obstetric practice patterns may have contributed to countries with lower or higher intervention rates.

It is difficult to establish which variables may have been affected by quality issues which may have biased the results. We found some remarkable and unexpected incidences for some variables. It is unlikely, for instance, that in most countries no cases of maternal deaths occurred within the first 7 postpartum days. It is known that maternal mortality is underreported, that definitions differ across countries, and that in some countries, separate registers exist to record maternal mortality and many national registers do not register maternal mortality until 42 days [21,22]. Previous studies recommended that increased attention should be paid to registering maternal morbidity more accurately [23,24]. In addition, low incidences of Apgar score < 7 in Malta and Chile and PPH in Malta may be a result of underreporting [25,26]. These problems limit interpretability of correlations between intervention rates and adverse outcome rates. Despite existing recommendations on indicator definitions, actions are still required to improve the registration of adverse outcomes across countries [25,26]. Last, the diagnosis of OASI is subjective and subject to the knowledge and experience of the care provider. High OASI rates can be a signal of overdiagnosis [27], whereas low rates may indicate underdiagnosis [28]. Hence, uniform means of diagnosing OASI across countries are required.

In eight countries, missing data were not recorded as a separate category within a variable, which may have led to underestimations of incidences in these countries. This problem has previously been described [16] but is still present in Finland, Sweden, Norway, Denmark, Iceland, Ireland, the state of Hesse (Germany), Malta, and Chile. This may have particularly influenced population characteristics and adverse outcomes and, to a lesser extent, intervention rates.

### Implications and recommendations

#### Adverse outcomes and their correlations

Our results indicate a correlation between countries having a higher rate of spontaneous vaginal births among nulliparous women and a higher rate of OASI in vaginal births. This may be explained by the fact that women with a higher risk of sphincter rupture (e.g., carrying macrosomic babies) may give birth vaginally in these countries but by CS in other countries. At the population level, the negative impact of increasing CS rates on women’s health has to be balanced against the possible benefits of reducing the generally low rate of OASI even further. Implementing interventions with the least possible harm to prevent the occurrence of OASI is recommended—for example, the use of warm compresses during the second stage of labour, which is still not used in many cases despite evidence of beneficial effect [29].

Rates of epidural anaesthesia among nulliparous women and rates of spontaneous vaginal births among multiparous women were positively correlated with rates of Apgar score < 7 at 5 minutes. Experimental studies did not show a causal relationship between epidural anaesthesia and low Apgar scores [30], but observational studies are not consistent in reporting correlations between epidural anaesthesia and Apgar score < 7 [31,32]. Besides, doses of epidural anaesthetics were not recorded and could have had some influence, and Apgar score may be underreported in some countries. Whereas CS can lead to improved perinatal outcomes when medically indicated, it may not improve perinatal outcomes when performed without medical indication [33]. Previous literature does not suggest that high rates of CS result in better perinatal outcomes [34]. Our results, suggesting that lower rates of epidural anaesthesia and spontaneous vaginal births on a national level may be negatively correlated with an Apgar score < 7, should be interpreted with caution. Further research is required to investigate these correlations.

Country policies, attitudes, and uptake of termination of pregnancy (TOP), both elective and for fetal complications, vary between countries. TOPs are more common among mothers who have unplanned pregnancies, are in unfavourable socioeconomic situations, are younger, and have major congenital anomalies, all of which are associated with adverse obstetric and fetal outcomes. In this study, characteristics of pregnancies ending in TOP were not included. Although variation in perinatal mortality rates was statistically not significant, it is conceivable that countries where TOP is restricted or illegal in 2013 (such as Ireland, Malta, and Chile) will experience higher rates of perinatal mortality.

#### Childbirth interventions

Adjustments for ethnicity, additional to adjustments for parity and maternal age, changed the ORs for augmentation of labour, episiotomy for vaginal births, and OASI. Countries with higher rates of women with a minority ethnicity or with a higher BMI had lower rates of episiotomy and higher rates of augmentation of labour, which led to changes in rankings. Our results indicate that BMI and ethnicity are explanatory factors in intercountry variation of augmentation of labour and episiotomy, but other population characteristics do not explain the variation, and population characteristics do not explain variation in other interventions or adverse outcomes. Besides, the variation in perinatal mortality rates did not reach statistical significance. Our study shows that univariable analyses and multivariable analyses with adjustments for population characteristics are essential when comparing rates of interventions and adverse outcome between countries.

Although the rates of spontaneous vaginal birth were positively correlated with OASI rates, we did not find a correlation between the rates of instrumental vaginal birth and OASI rates. This can be explained by the very low instrumental birth rates in some countries. However, if instrumental births were performed more frequently, the excessively high rate of CS in these countries is likely to be reduced considerably. This is supported by other studies that found a negative correlation between instrumental birth rates and CS rates [35]. In countries with the lowest instrumental birth rates, the highest prelabour and overall CS rates were found. Besides, the CS rate in the USA and Chile was higher among multiparous women than among nulliparous women. This indicates that percentages of vaginal birth after CS (VBAC) are low. Both instrumental vaginal births and VBACs are important in promoting vaginal births and decreasing CS rates [35,36].

Our finding of higher intervention rates in countries with very low incidences of births beyond 42 weeks’ gestation should be interpreted with some caution, because the methods for assessing gestational age may differ between countries. Nevertheless, this result suggests that intervening in the process of pregnancy and childbirth is more common in some countries, without this being explained by differences in population characteristics. We expected that higher rates of prelabour CS would be correlated with lower rates of emergency intrapartum CS, but we found the opposite. Besides, higher rates of induction of labour were not correlated with lower prelabour CS rates. As a result, in some countries, just over half of all women had a spontaneous onset of labour, as opposed to 80% in other countries.

Factors that may be associated with decisions to intervene in labour include economic, psychological, organisational, and cultural [37]. First of all, differences in healthcare between countries are influenced by differences in the financial models of reimbursing healthcare [37]. Financial incentives play an important role in, for example, CS rates [38].

Besides social history, developments in attitudes towards childbirth over time and differences in education of care providers [39–41] influence the working environment, as well as care providers’ attitudes towards physiological birth and risk management [42–44]. A working environment where childbirth is seen as a very risky event is conducive to intervening more [42–44]. This is endorsed by our findings that indicate differences in the birth culture—namely, high CS rates in countries with mainly low instrumental vaginal birth rates; higher intervention rates in countries with lower rates of births beyond 42 weeks; higher rates of interventions in countries with more hospital births; lower rates of episiotomy in countries with more spontaneous vaginal births; and a positive correlation between prelabour CS and emergency intrapartum CS rates. A major focus on prevention of adverse outcomes and a birth culture in which childbirth is seen as a very risky event can lead to higher rates of interventions [42–44]. In cultures with generally lower intervention rates, higher rates of instrumental vaginal births and VBACs and more advanced gestational ages at birth were found [35,36]. Birth cultures and values in the wider society play an important role in intercountry variation [39–41] and influence women’s preferences as well. On the other hand, women’s preferences have less influence on the rate of episiotomy, which also varies considerably across countries. Although the episiotomy rate is declining worldwide, the decline has been faster in Sweden, Denmark, and Iceland [45]. This has resulted in much lower episiotomy rates in these countries compared with many others.

Views and attitudes towards childbirth, as well as the history of maternal healthcare, can also, to an extent, be reflected in national guidelines. Differences in national guidelines can lead to variations in intervention rates. However, the existence of comparable guidelines between countries has not yet led to equal quality of care [46]. Studies on regional variations in countries with national guidelines also showed large variations between regions making use of the same guidelines [47,48]. This may be explained by differences in adherence to, and interpretation of, international and national guidelines. Implementation of international guidelines is an important step in achieving optimal use of interventions and equal quality of care for all women. This effort has been assisted by the release of the recommendations in ‘Intrapartum care for a positive childbirth experience’ by WHO [49].

This study highlights strikingly high and low intervention rates and contributes to the debate on under- and overuse of interventions [2,4,49]. The major international variation in the use of childbirth interventions found in our study is consistent with previous studies [7–10] and has been the subject of concern for years [10]. Recent studies advocate a paradigm shift towards reducing unnecessarily high intervention rates and encouraging implementation of supportive preventive care [50–52]. Overuse of interventions is not only a problem in high-income countries; inequities in care for women occur in certain subpopulations in countries of all income levels, resulting in some women receiving too few interventions while others in the same country receive interventions too frequently [2]. Further studies should focus on the barriers to reducing unnecessarily high intervention rates, reducing inequities in maternal healthcare, reporting differences in indications for childbirth interventions, and examining the influence of the healthcare system on rates of interventions and care processes. The evidence from this international study and other studies should be used to implement recommendations for national data registration and to improve comparability of international data [24].

To make further research possible, national data from all countries should be uniformly recorded and available for research, without being hindered by the General Data Protection Regulation (GDPR) 2018, with agreements on the standards used to define indicators. Besides, it is recommended that medical indications are recorded in routinely collected data with sufficient detail to ensure valid comparisons [53]. Moreover, a crucial step in the improvement of maternity and perinatal healthcare is that care providers and policy makers implement evidence-based interventions to decrease inequity in maternal healthcare [2].

In conclusion, this study found large variation in the use of interventions that cannot be explained solely by population and clinical differences, with the largest variation being for augmentation of labour, pain relief, episiotomy, instrumental vaginal birth, and CS. Although adjustments for population characteristics did not result in large changes, two variable rankings changed, indicating that adjustments are important when comparing rates between countries. No statistically significant variation was found for perinatal mortality. Higher rates of interventions were correlated with lower rates of births beyond 42 weeks’ of gestation, and higher rates of prelabour CS were correlated with higher rates of emergency intrapartum CS. This study contributes to the debate about optimal rates of childbirth interventions and shows limitations in the quality of the available national data. To facilitate a valid comparison of healthcare between countries, national data collection needs to be improved and standardised. Implementation of evidence-based guidelines in practice is a crucial step in optimising the use of interventions. This step is urgently needed to reduce intrapartum interventions that do not have beneficial maternal or neonatal outcomes and improve the quality of maternity care around the world.

## Supporting information

S1 STROBE Checklist STROBE, Strengthening the Reporting of Observational Studies in Epidemiology. (DOCX)

S1 Table National and data characteristics in 2013 by country. (DOCX)

S2 Table Crude ORs of childbirth interventions by country, compared with the weighted mean, with 99% CIs. CI, confidence interval; OR, odds ratio. (DOCX)

S3 Table Crude ORs and adjusted ORs of adverse neonatal and maternal outcomes by country, compared with the weighted mean, with 99% CIs. CI, confidence interval; OR, odds ratio. (DOCX)

S4 Table Crude ORs and for parity and maternal age–adjusted ORs of childbirth interventions by country, compared with the weighted mean, with 99% CIs. CI, confidence interval; OR, odds ratio. (DOCX)

S5 Table Crude ORs and for parity, maternal age–and ethnicity-adjusted ORs of childbirth interventions by country, compared with the weighted mean, with 99% CIs. CI, confidence interval; OR, odds ratio. (DOCX)

S6 Table Crude ORs and for parity, maternal age–and maternal body mass index–adjusted ORs of childbirth interventions by country, compared with the weighted mean, with 99% CIs. CI, confidence interval; OR, odds ratio. (DOCX)

S7 Table Crude ORs and for parity, maternal age–and education-adjusted ORs of childbirth interventions by country, compared with the weighted mean, with 99% CIs. CI, confidence interval; OR, odds ratio. (DOCX)

S8 Table Crude ORs and for parity, maternal age–and birth weight–adjusted ORs of childbirth interventions by country, compared with the weighted mean, with 99% CIs. CI, confidence interval; OR, odds ratio. (DOCX)

S1 Data Statement Details on data statements per country. (PDF)

S1 Fig Interventions among nulliparous women with a correlation with hospital birth. (TIF)

S2 Fig Interventions (nulliparous women) and adverse outcomes with a correlation with epidural anaesthesia (nulliparous women). (TIF)

S3 Fig Interventions (nulliparous women) and adverse outcomes with a correlation with spontaneous vaginal birth (nulliparous women). (TIF)

S4 Fig Interventions (multiparous women) and adverse outcomes with a correlation with spontaneous vaginal birth (multiparous women). (TIF)

## Abbreviations

aOR: adjusted odds ratio
BMI: body mass index
CI: confidence interval
COST: European Cooperation in Science and Technology
CS: cesarean section
GDPR: General Data Protection Regulation
OASI: obstetric anal sphincter injury
OR: odds ratio
PPH: postpartum haemorrhage ≥ 1,000 ml
STROBE: Strengthening the Reporting of Observational Studies in Epidemiology
TOP: termination of pregnancy
VBAC: vaginal birth after cesarean section
WHO: World Health Organization

## References

[1] Woods R. Long-term trends in fetal mortality: implications for developing countries. Bull World Health Organ. 2008;86(6):460–6. 10.2471/BLT.07.043471 18568275 PMC2647461

[2] Miller S, Abalos E, Chamillard M, Ciapponi A, Colaci D, Comande D, et al Beyond too little, too late and too much, too soon: a pathway towards evidence-based, respectful maternity care worldwide. Lancet. 2016;388(10056):2176–92. 10.1016/S0140-6736(16)31472-6 27642019

[3] The Lancet Ending Preventable Stillbirths study group. Ending preventable stillbirths. The Lancet [Internet]. 2016 [cited 2019 Jul 15]. Available from: http://www.thelancet.com/pb/assets/raw/Lancet/stories/series/stillbirths2016-exec-summ.pdf.

[4] Brownlee S, Chalkidou K, Doust J, Elshaug AG, Glasziou P, Heath I, et al Evidence for overuse of medical services around the world. Lancet. 2017;390(10090):156–68. 10.1016/S0140-6736(16)32585-5 28077234 PMC5708862

[5] Gibbons L, Belizán J, Lauer J, Betrán A, Merialdi M, Althabe F. The Global Numbers and Costs of Additionally Needed and Unnecessary Caesarean Sections Performed per Year: Overuse as a Barrier to Universal Coverage. World Health Report Background Paper. 2010;30:1–31.

[6] Gray M, Jani A. Promoting Triple Value Healthcare in Countries with Universal Healthcare. Healthc Pap. 2016;15(3):42–8. 27009586

[7] EURO-PERISTAT Project with SCPE and EUROCAT. European Perinatal Health Report. Health and care of pregnant women and babies in Europe in 2010. EURO-PERISTAT; 2013.

[8] Notzon FC. International differences in the use of obstetric interventions. JAMA. 1990;263(24):3286–91. 2348539

[9] Blondel B, Alexander S, Bjarnadottir RI, Gissler M, Langhoff-Roos J, Novak-Antolic Z, et al Variations in rates of severe perineal tears and episiotomies in 20 European countries: a study based on routine national data in Euro-Peristat Project. Acta Obstet Gynecol Scand. 2016;95(7):746–54. 10.1111/aogs.12894 26958827

[10] Betran AP, Merialdi M, Lauer JA, Bing-Shun W, Thomas J, Van LP, et al Rates of caesarean section: analysis of global, regional and national estimates. Paediatr Perinat Epidemiol. 2007;21(2):98–113. 10.1111/j.1365-3016.2007.00786.x 17302638

[11] Seijmonsbergen-Schermers A, van den Akker T, Beeckman K, Bogaerts A, Barros M, Janssen P, et al Variations in childbirth interventions in high-income countries: protocol for a multinational cross-sectional study. BMJ Open. 2018;8(1):e017993 10.1136/bmjopen-2017-017993 29326182 PMC5780680

[12] EU Birth Research Project. A COST Action website detailing research into birth practices. Information about the EU Birth Research Project. 2018 [cited 2019 Jul 15]. Available from: https://eubirthresearch.wordpress.com.

[13] Sultan AH, Thakar R. Lower genital tract and anal sphincter trauma. Best Pract Res Clin Obstet Gynaecol. 2002;16(1):99–115. 10.1053/beog.2002.0258 11866500

[14] Van Buuren S, Groothuid-Oudshoorn K. Multivariate Imputation by Chained Equations in R. Journal of Statistical Software. 2001;45(3):1–67.

[15] The BMJ. 11. Correlation and regression. The BMJ [Internet]. 2018 [2019 Jul 15]. Available from: http://www.bmj.com/about-bmj/resources-readers/publications/statistics-square-one/11-correlation-and-regression.

[16] Gissler M, Mohangoo AD, Blondel B, Chalmers J, Macfarlane A, Gaizauskiene A, et al Perinatal health monitoring in Europe: results from the EURO-PERISTAT project. Inform Health Soc Care. 2010;35(2):64–79. 10.3109/17538157.2010.492923 20726736

[17] EURO-PERISTAT. Euro-Peristat list of indicators, updated 2012. [cited 2019 Jul 15]. Available from: https://www.europeristat.com/images/doc/updated%20indicator%20list.pdf.

[18] Zeitlin J, Wildman K, Breart G, Alexander S, Barros H, Blondel B, et al Selecting an indicator set for monitoring and evaluating perinatal health in Europe: criteria, methods and results from the PERISTAT project. Eur J Obstet Gynecol Reprod Biol. 2003;111 Suppl 1:S5–s14. 14642316 10.1016/j.ejogrb.2003.09.002

[19] Reproductive Maternal and Child Health European Regional office World Health Organization. Definitions and indicators in family planning maternal & child health and reproductive health. Used in the WHO regional office for Europe. 2001 [cited 2019 Jul 15]. Available from: https://apps.who.int/iris/bitstream/handle/10665/108284/E68459.pdf?sequence=1&isAllowed=y.

[20] EURO-PERISTAT. EURO-PERISTAT indicators of perinatal health 2018 [2019 Jul 15]. Available from: https://www.europeristat.com/our-indicators/indicators-of-perinatal-health.html#footer.

[21] Bouvier-Colle MH, Mohangoo AD, Gissler M, Novak-Antolic Z, Vutuc C, Szamotulska K, et al What about the mothers? An analysis of maternal mortality and morbidity in perinatal health surveillance systems in Europe. BJOG. 2012;119(7):880–9. 10.1111/j.1471-0528.2012.03330.x 22571748 PMC3472023

[22] Deneux-Tharaux C, Berg C, Bouvier-Colle MH, Gissler M, Harper M, Nannini A, et al Underreporting of pregnancy-related mortality in the United States and Europe. Obstet Gynecol. 2005;106(4):684–92. 10.1097/01.AOG.0000174580.24281.e6 16199622

[23] Sauvegrain P, Chantry AA, Chiesa-Dubruille C, Keita H, Goffinet F, Deneux-Tharaux C. Monitoring quality of obstetric care from hospital discharge databases: A Delphi survey to propose a new set of indicators based on maternal health outcomes. PLoS ONE. 2019;14(2):e0211955 10.1371/journal.pone.0211955 30753232 PMC6372226

[24] Schaap T, Bloemenkamp K, Deneux-Tharaux C, Knight M, Langhoff-Roos J, Sullivan E, et al Defining definitions: a Delphi study to develop a core outcome set for conditions of severe maternal morbidity. BJOG. 2019;126(3):394–401. 10.1111/1471-0528.14833 28755459

[25] Lain SJ, Roberts CL, Hadfield RM, Bell JC, Morris JM. How accurate is the reporting of obstetric haemorrhage in hospital discharge data? A validation study. Aust N Z J Obstet Gynaecol. 2008;48(5):481–4. 10.1111/j.1479-828X.2008.00910.x 19032664

[26] O'Donnell CP, Kamlin CO, Davis PG, Carlin JB, Morley CJ. Interobserver variability of the 5-minute Apgar score. J Pediatr. 2006;149(4):486–9. 10.1016/j.jpeds.2006.05.040 17011319

[27] Sioutis D, Thakar R, Sultan AH. Overdiagnosis and rising rate of obstetric anal sphincter injuries (OASIS): time for reappraisal. Ultrasound Obstet Gynecol. 2017;50(5):642–7. 10.1002/uog.17306 27643513

[28] Ginath S, Mizrachi Y, Bar J, Condrea A, Kovo M. Obstetric Anal Sphincter Injuries (OASIs) in Israel: A Review of the Incidence and Risk Factors. Rambam Maimonides Med J. 2017;8(2). 10.5041/RMMJ.10295 PMC5415364 28467760

[29] Aasheim V, Nilsen ABV, Reinar LM, Lukasse M. Perineal techniques during the second stage of labour for reducing perineal trauma. Cochrane Database Syst Rev. 2017;6:CD006672 10.1002/14651858.CD006672.pub3 28608597 PMC6481402

[30] Anim-Somuah M, Smyth RM, Cyna AM, Cuthbert A. Epidural versus non-epidural or no analgesia for pain management in labour. Cochrane Database Syst Rev. 2018;5:Cd000331. 10.1002/14651858.CD000331.pub4 PMC6494646 29781504

[31] Tornell S, Ekeus C, Hultin M, Hakansson S, Thunberg J, Hogberg U. Low Apgar score, neonatal encephalopathy and epidural analgesia during labour: a Swedish registry-based study. Acta Anaesthesiol Scand. 2015;59(4):486–95. 10.1111/aas.12477 25683882

[32] Hincz P, Podciechowskil L, Grzesiak M, Horzelski W, Wilczyflski J. Epidural analgesia during labor: a retrospective cohort study on its effects on labour, delivery and neonatal outcome. Ginekol Pol. 2014;85(12):923–8. 25669061

[33] Fagerberg MC, Marsal K, Kallen K. Neonatal outcome after trial of labor or elective cesarean section in relation to the indication for the previous cesarean delivery. Acta Obstet Gynecol Scand. 2013;92(10):1151–8. 10.1111/aogs.12202 23782390

[34] Kupari M, Talola N, Luukkaala T, Tihtonen K. Does an increased cesarean section rate improve neonatal outcome in term pregnancies? Arch Gynecol Obstet. 2016;294(1):41–6. 10.1007/s00404-015-3942-4 26573011

[35] Plevani C, Incerti M, Del Sorbo D, Pintucci A, Vergani P, Merlino L, et al Cesarean delivery rates and obstetric culture—an Italian register-based study. Acta Obstet Gynecol Scand. 2017;96(3):359–65. 10.1111/aogs.13063 27869984

[36] Davis G, Fleming T, Ford K, Mouawad MR, Ludlow J. Caesarean section at full cervical dilatation. Aust N Z J Obstet Gynaecol. 2015;55(6):565–71. 10.1111/ajo.12374 26223774

[37] Reschovsky JD, Rich EC, Lake TK. Factors Contributing to Variations in Physicians' Use of Evidence at The Point of Care: A Conceptual Model. J Gen Intern Med. 2015;30 Suppl 3:S555–61. 26105673 10.1007/s11606-015-3366-7 PMC4512965

[38] Hoxha I, Syrogiannouli L, Luta X, Tal K, Goodman DC, da Costa BR, et al Caesarean sections and for-profit status of hospitals: systematic review and meta-analysis. BMJ Open. 2017;7(2):e013670 10.1136/bmjopen-2016-013670 28213600 PMC5318567

[39] Healy S, Humphreys E, Kennedy C. Midwives' and obstetricians' perceptions of risk and its impact on clinical practice and decision-making in labour: An integrative review. Women Birth. 2016;29(2):107–16. 10.1016/j.wombi.2015.08.010 26363668

[40] Mead MM, Kornbrot D. The influence of maternity units' intrapartum intervention rates and midwives' risk perception for women suitable for midwifery-led care. Midwifery. 2004;20(1):61–71. 10.1016/S0266-6138(03)00054-8 15020028

[41] Cromi A, Bonzini M, Uccella S, Serati M, Bogani G, Pozzo N, et al Provider contribution to an episiotomy risk model. J Matern Fetal Neonatal Med. 2015;28(18):2201–6. 10.3109/14767058.2014.982087 25380033

[42] Toohill J, Sidebotham M, Gamble J, Fenwick J, Creedy DK. Factors influencing midwives' use of an evidenced based Normal Birth Guideline. Women Birth. 2017;30(5):415–23. 10.1016/j.wombi.2017.03.008 28434673

[43] Carolan-Olah M, Kruger G, Garvey-Graham A. Midwives' experiences of the factors that facilitate normal birth among low risk women at a public hospital in Australia. Midwifery. 2015;31(1):112–21. 10.1016/j.midw.2014.07.003 25132098

[44] Zinsser LA, Stoll K, Gross MM. Midwives' attitudes towards supporting normal labour and birth—A cross-sectional study in South Germany. Midwifery. 2016;39:98–102. 10.1016/j.midw.2016.05.006 27321726

[45] Laine K, Gissler M, Pirhonen J. Changing incidence of anal sphincter tears in four Nordic countries through the last decades. Eur J Obstet Gynecol Reprod Biol. 2009;146(1):71–5. 10.1016/j.ejogrb.2009.04.033 19482405

[46] Macfarlane AJ, Blondel B, Mohangoo AD, Cuttini M, Nijhuis J, Novak Z, et al Wide differences in mode of delivery within Europe: risk-stratified analyses of aggregated routine data from the Euro-Peristat study. BJOG. 2016;123(4):559–68. 10.1111/1471-0528.13284 25753683

[47] Seijmonsbergen-Schermers AE, Zondag DC, Nieuwenhuijze M, Van den Akker T, Verhoeven C, Geerts C, et al Regional Variations in Interventions in Childbirth in the Netherlands: a nationwide study. BMC Pregnancy Childbirth. 2018;18(192). 10.1186/s12884-017-1653-5 29855270 PMC5984340

[48] Seijmonsbergen-Schermers AE, Zondag DC, Nieuwenhuijze M, Van den Akker T, Verhoeven C, Geerts C, et al Regional variations in childbirth interventions and their correlations with adverse outcomes, birthplace and care provider: a nationwide explorative study. PLoS ONE 2020;15(3):e0229488 10.1371/journal.pone.0229488 32134957 PMC7058301

[49] World Health Organization. WHO recommendations: Intrapartum care for a positive childbirth experience. Geneva: World Health Organization; 2018. 30070803

[50] Betran AP, Temmerman M, Kingdon C, Mohiddin A, Opiyo N, Torloni MR, et al Interventions to reduce unnecessary caesarean sections in healthy women and babies. Lancet. 2018;392(10155):1358–68. 10.1016/S0140-6736(18)31927-5 30322586

[51] Boerma T, Ronsmans C, Melesse DY, Barros AJD, Barros FC, Juan L, et al Global epidemiology of use of and disparities in caesarean sections. Lancet. 2018;392(10155):1341–8. 10.1016/S0140-6736(18)31928-7 30322584

[52] Sandall J, Tribe RM, Avery L, Mola G, Visser GH, Homer CS, et al Short-term and long-term effects of caesarean section on the health of women and children. Lancet. 2018;392(10155):1349–57. 10.1016/S0140-6736(18)31930-5 30322585

[53] Vogel JP, Betran AP, Vindevoghel N, Souza JP, Torloni MR, Zhang J, et al Use of the Robson classification to assess caesarean section trends in 21 countries: a secondary analysis of two WHO multicountry surveys. Lancet Glob Health. 2015;3(5):e260–70. 10.1016/S2214-109X(15)70094-X 25866355

