# Supplementary material for: Variations in use of childbirth interventions in 13 high-income countries: A multinational cross-sectional study
Source: PLoS Med. 2020 May 22;17(5):e1003103. doi: 10.1371/journal.pmed.1003103 (PMC7244098; doi:10.1371/journal.pmed.1003103)
Supplement: S1 STROBE Checklist — (DOCX) [file pmed.1003103.s001.docx]

STROBE Statement—Checklist of items that should be included in reports of ***cross-sectional studies***

|  | Item No | Recommendation | Section and paragraph No |
| --- | --- | --- | --- |
| **Title and abstract** | 1 | (*a*) Indicate the study’s design with a commonly used term in the title or the abstract | Title page |
|  |  | (*b*) Provide in the abstract an informative and balanced summary of what was done and what was found | Abstract |
| Introduction | | | |
| Background/rationale | 2 | Explain the scientific background and rationale for the investigation being reported | Introduction, first and second paragraph |
| Objectives | 3 | State specific objectives, including any prespecified hypotheses | Introduction, second paragraph |
| Methods | | | |
| Study design | 4 | Present key elements of study design early in the paper | Methods, first paragraph |
| Setting | 5 | Describe the setting, locations, and relevant dates, including periods of recruitment, exposure, follow-up, and data collection | Methods under heading ‘Data collection’ |
| Participants | 6 | (*a*) Give the eligibility criteria, and the sources and methods of selection of participants | Methods under heading ‘Data collection’ |
| Variables | 7 | Clearly define all outcomes, exposures, predictors, potential confounders, and effect modifiers. Give diagnostic criteria, if applicable | Methods under headings ‘Independent and dependant variables’ and ‘Analysis and missing data’ |
| Data sources/ measurement | 8* | For each variable of interest, give sources of data and details of methods of assessment (measurement). Describe comparability of assessment methods if there is more than one group | Methods under headings ‘Independent and dependant variables’ and ‘Analysis and missing data’ |
| Bias | 9 | Describe any efforts to address potential sources of bias | Methods under headings ‘Analysis and missing data’ and ‘Quality assessment’ |
| Study size | 10 | Explain how the study size was arrived at | Methods under heading ‘Data collection’ |
| Quantitative variables | 11 | Explain how quantitative variables were handled in the analyses. If applicable, describe which groupings were chosen and why | Methods under headings ‘Independent and dependant variables’ and ‘Analysis and missing data’ |
| Statistical methods | 12 | (*a*) Describe all statistical methods, including those used to control for confounding | Methods under heading ‘Analysis and missing data’ |
|  |  | (*b*) Describe any methods used to examine subgroups and interactions | Methods under heading ‘Analysis and missing data’ |
|  |  | (*c*) Explain how missing data were addressed | Methods under heading ‘Analysis and missing data’ |
|  |  | (*d*) If applicable, describe analytical methods taking account of sampling strategy | Methods under heading ‘Data collection’ |
|  |  | (*e*) Describe any sensitivity analyses | NA |
| Results | | | |
| Participants | 13* | (a) Report numbers of individuals at each stage of study—eg numbers potentially eligible, examined for eligibility, confirmed eligible, included in the study, completing follow-up, and analysed | Results under heading ’Included countries and missing data’ |
|  |  | (b) Give reasons for non-participation at each stage | Results under heading ’Included countries and missing data’ |
|  |  | (c) Consider use of a flow diagram | NA |
| Descriptive data | 14* | (a) Give characteristics of study participants (eg demographic, clinical, social) and information on exposures and potential confounders | Results under heading ‘Variations in population and birth characteristics’ |
|  |  | (b) Indicate number of participants with missing data for each variable of interest | Tables 1 to 5 |
| Outcome data | 15* | Report numbers of outcome events or summary measures | Tables 1 to 5 and Results under headings ‘Variations in intervention rates’ and ‘Variations in adverse outcomes’ |
| Main results | 16 | (*a*) Give unadjusted estimates and, if applicable, confounder-adjusted estimates and their precision (eg, 95% confidence interval). Make clear which confounders were adjusted for and why they were included | Tables in Supporting Information S2 to S8 and Results under heading ‘Adjustments for population characteristics’ |
|  |  | (*b*) Report category boundaries when continuous variables were categorized | NA |
|  |  | (*c*) If relevant, consider translating estimates of relative risk into absolute risk for a meaningful time period | NA |
| Other analyses | 17 | Report other analyses done—eg analyses of subgroups and interactions, and sensitivity analyses | Tables 6 and 7 and Results under headings ‘Variations in intervention rates’ and ‘Variations in adverse outcomes’ |
| Discussion | | | |
| Key results | 18 | Summarise key results with reference to study objectives | Discussion, first paragraph |
| Limitations | 19 | Discuss limitations of the study, taking into account sources of potential bias or imprecision. Discuss both direction and magnitude of any potential bias | Discussion under heading ‘Limitations and strengths’ |
| Interpretation | 20 | Give a cautious overall interpretation of results considering objectives, limitations, multiplicity of analyses, results from similar studies, and other relevant evidence | Discussion under headings ‘Adverse outcomes and their correlations’ and ‘Interpretation and further research’ |
| Generalisability | 21 | Discuss the generalisability (external validity) of the study results | Discussion under headings ‘Limitations and strengths’ |
| Other information | | | |
| Funding | 22 | Give the source of funding and the role of the funders for the present study and, if applicable, for the original study on which the present article is based | Declarations under heading ‘Funding’ |

*Give information separately for exposed and unexposed groups.

**Note:** An Explanation and Elaboration article discusses each checklist item and gives methodological background and published examples of transparent reporting. The STROBE checklist is best used in conjunction with this article (freely available on the Web sites of PLoS Medicine at http://www.plosmedicine.org/, Annals of Internal Medicine at http://www.annals.org/, and Epidemiology at http://www.epidem.com/). Information on the STROBE Initiative is available at www.strobe-statement.org.
