# Supplementary material for: Variations in use of childbirth interventions in 13 high-income countries: A multinational cross-sectional study
Source: PLoS Med. 2020 May 22;17(5):e1003103. doi: 10.1371/journal.pmed.1003103 (PMC7244098; doi:10.1371/journal.pmed.1003103)
Supplement: S1 Table — (DOCX) [file pmed.1003103.s002.docx]

**S1 Table. National and data characteristics in 2013 by country**

| *** | **FIN** | **SWE** | **NOR** | **DNK** | **ISL** | **IRL** | **ENG** | **NLD** | **BEL** | **HESSE** | **MLT** | **USA** | **CHL** |
| --- | --- | --- | --- | --- | --- | --- | --- | --- | --- | --- | --- | --- | --- |
| **Total number of inhabitants, *n*** | 5,  438,972 | 9,  600,379 | 5,  079,623 | 5,  614,932 | 323,764 | 4,  598,294 | 53,  865,800 | 16,  804,432 | 11,  182,817 | 6,  045,425 | 423,374 | 316,  427,395 | 17,  575,833 |
| **National number of births *$*, *n*** | 58,525 | 113,593 | 59,982 | 56,584 | 4,236 | 69,267 | 664,517 | 168,553 | 124,862 | 52,185 | 4,149 | 3,932,181 | 242,005 |
| **Proportion births in study *$*, *%*** | 92.8% | 92.1% | 91.6% | 89.6% | 94.1% | 90.4% | 61.8% | 90.6% | 90.4% | 85.7% | 91.1% | 89.0% | 71.6% |
| **Gross national income (GNI) per capita in 2013** | $ 41,430 | $ 60,910 | $ 67,730 | $ 48,250 | $ 41,480 | $ 41,100 | $ 38,780 | $ 49,390 | $ 44,210 | $ 45,972 | $ 29,520 | $ 53,960 | $ 21,690 |
| **Source of the data *#*** | B | B | B | ABC | B | C | ABC | B | ABG | C | B | A | BCD |
| **Health care system (public and/or private)** | Public | Public | Mainly public, small amount private | Public | Mainly public, small amount private | Public (±80%) and private (±20%) | Mainly public, small amount private | Publicly regulated with private insurers | Mainly public, small amount private | Public and private | Public, very small amount private | Public and private | Public (±80%) and private (±20%) |

Total number of inhabitants, national number of births and GNI: data from the World Bank or national statistics; for England: data from the office of national statistics and the OECD, and the GNI is of the United Kingdom.

* Meaning of the abbreviations: FIN = Finland; NOR = Norway; DNK = Denmark; ISL = Iceland; IRL = Ireland; ENG = England; NLD = The Netherlands; BEL=Belgium; HESSE = Hesse (state of Germany); MLT = Malta; USA = United States of America; CHL = Chile

*$* National number of births: including multiple and preterm births; Proportion births in dataset: multiple and preterm births are excluded in this study.

*#* Source of the data: A=civil registration; B=medical birth register or child health system; C=hospital discharge system; D=perinatal survey; E=confidential enquiry; F=other routine surveys; G=linked data source; H=other.
