# Supplementary material for: Variations in use of childbirth interventions in 13 high-income countries: A multinational cross-sectional study
Source: PLoS Med. 2020 May 22;17(5):e1003103. doi: 10.1371/journal.pmed.1003103 (PMC7244098; doi:10.1371/journal.pmed.1003103)
Supplement: S2 Table — (DOCX) [file pmed.1003103.s003.docx]

**S2 Table. Crude ORs of childbirth interventions by country in 2013, compared to the weighted mean*, with 99% CIs**

|  | **FIN** | **SWE** | **NOR** | **DNK** | **ISL** | **IRL** | **ENG** | **NLD** | **BEL** | **HESSE** | **MLT** | **USA** | **CHL** |
| --- | --- | --- | --- | --- | --- | --- | --- | --- | --- | --- | --- | --- | --- |
| **Total *n*** | 54,310 |  | 54,951 | 50,692 | 3,987 | 62,613 | 410,366 | 152,644 | 112,907 | 44,722 | 3,781 | 3,500,397 | 173,477 |
| **Births at ≥42 weeks**  Crude OR [99% CI] | 4.48  [3.91-5.14] | 6.60  [5.78-7.53] | 3.85  [3.35-4.42] | 1.76  [1.51-2.04] | 2.02  [1.52-2.69] | - | 4.22  [3.71-4.81] | 1.34  [1.17-1.54] | 0.23  [0.19-0.28] | 0.54  [0.44-0.65] | 0.07  [0.02-0.28] | 0.43  [0.37-0.48] | 0.11  [0.09-0.14] |
| **Spontaneous onset of labour**  Crude OR [99% CI] | 1.40  [1.37-1.44] | 2.11  [2.16-2.26] | 1.69  [1.65-1.74] | 0.81  [0.79-0.83] | 1.32  [1.21-1.44] | 0.68  [0.67-0.70] | 0.95  [0.94-0.96] | 0.93  [0.92-0.95] | 0.83  [0.82-0.85] | 0.98  [0.96-1.01] | 0.59  [0.54-0.64] | 0.61  [0.60-0.62] | - |
| **Induction of labour**  Crude OR [99% CI] | 0.89  [0.86-0.91] | 0.41  [0.40-0.42] | 0.70  [0.68-0.72] | 1.31  [1.28-1.35] | 0.96  [0.88-1.05] | 1.31  [1.28-1.34] | 1.03  [1.01-1.04] | 1.34  [1.32-1.37] | 1.30  [1.28-1.33] | 0.87  [0.85-0.90] | 1.49  [1.37-1.62] | 1.03  [1.01-1.04] | - |
| **Prelabour CS**  Crude OR [99% CI] | 0.57  [0.54-0.59] | 0.77  [0.75-0.80] | 0.59  [0.56-0.61] | 1.09  [1.05-1.13] | 0.56  [0.48-0.66] | 1.57  [1.52-1.62] | 1.05  [1.03-1.08] | 0.67  [0.65-0.69] | 1.02  [0.99-1.05] | 1.42  [1.37-1.48] | 1.69  [1.52-1.88] | 2.36  [2.32-2.41] | - |
| **Augmentation of labour**  Crude OR [99% CI] | 3.23  [3.14-3.32] | - | 1.61  [1.56-1.65] | 0.58  [0.56-0.60] | 0.50  [0.45-0.55] | 0.55  [0.53-0.57] | - | 0.96  [0.94-0.99] | - | - | - | 1.26  [1.24-1.29] | - |
| **Intrapartum use of oxytocin**  Crude OR [99% CI] | 2.03  [1.98-2.09] | - | 1.04  [1.01-1.07] | 0.64  [0.62-0.66] | 0.58  [0.54-0.63] | - | - | 1.26  [1.23-1.29] | - | - | - | - | - |
| **Artificial rupture of membranes**  Crude OR [99% CI] | 1.76  [1.71-1.81] | - | - | 0.58  [0.56-0.60] | 1.04  [0.98-1.11] | 0.94  [0.91-0.97] | - | - | - | - | - | - | - |
| **Any pain relief**  Crude OR [99% CI] | 2.44  [2.36-2.52] | 4.09  [3.96-4.22] | 0.89  [0.86-0.91] | 0.35  [0.34-0.36] | 1.09  [1.00-1.18] | - | - | 0.28  [0.28-0.29] | - | 0.37  [0.35-0.38] | 2.88  [2.57-3.23] | - | - |
| **Epidural**  Crude OR [99% CI] | 3.44  [3.36-3.53] | 0.71  [0.69-0.72] | 0.83  [0.81-0.85] | 0.61  [0.59-0.62] | 1.15  [1.06-1.24] | 1.49  [1.45-1.52] | 0.26  [0.25-0.26] | 0.37  [0.36-0.37] | 3.86  [3.78-3.94] | 0.56  [0.55-0.58] | 0.62  [0.56-0.68] | 3.74  [3.69-3.79] | - |
| **Other pharmacological pain relief**  Crude OR [99% CI] | 2.67  [2.60-2.75] | 2.71  [2.65-2.78] | 0.94  [0.91-0.97] | 0.34  [0.33-0.35] | 0.98  [0.91-1.06] | - | - | 0.28  [0.27-0.28] | - | 0.39  [0.38-0.41] | 4.06  [3.69-4.47] | - | - |
| **Episiotomy in vaginal births**  Crude OR [99% CI] | 1.35  [1.31-1.40] | 0.19  [0.18-0.20] | 1.12  [1.08-1.16] | 0.19  [0.18-0.21] | 0.66  [0.58-0.74] | 1.48  [1.43-1.53] | - | 1.85  [1.81-1.90] | 4.02  [3.91-4.12] | 1.44  [1.38-1.49] | 1.70  [1.53-1.88] | - | - |
| **Spontaneous vaginal birth**  Crude OR [99% CI] | 1.40  [1.36-1.43] | 1.50  [1.47-1.54] | 1.33  [1.30-1.37] | 1.17  [1.14-1.20] | 1.59  [1.45-1.74] | 0.60  [0.59-0.61] | 0.82  [0.80-0.83] | 1.42  [1.39-1.44] | 1.13  [1.11-1.15] | 0.71  [0.69-0.73] | 0.90  [0.82-0.97] | 0.89  0.88-0.90] | 0.44  [0.43-0.45] |
| **Instrumental vaginal birth**  Crude OR [99% CI] | 1.33  [1.27-1.38] | 1.12  [1.08-1.16] | 1.46  [1.40-1.52] | 1.24  [1.18-1.29] | 1.10  [0.96-1.27] | 2.28  [2.21-2.36] | 1.77  [1.73-1.81] | 1.19  [1.16-1.23] | 1.33  [1.29-1.37] | 0.88  [0.83-0.92] | 0.64  [0.54-0.77] | 0.46  [0.45-0.47] | 0.20  [0.19-0.21] |
| **Caesarean Section**  Crude OR [99% CI] | 0.61  [0.59-0.63] | 0.67  [0.65-0.68] | 0.60  [0.58-0.62] | 0.90  [0.88-0.93] | 0.56  [0.51-0.63] | 1.31  [1.28-1.35] | 1.05  [1.03-1.06] | 0.63  [0.62-0.64] | 0.81  [0.79-0.83] | 1.64  [1.60-1.69] | 1.39  [1.27-1.51] | 1.50  [1.48-1.52] | 3.34  [3.29-3.40] |
| **Emergency CS**  Crude OR [99% CI] | 0.88  [0.85-0.92] | 0.79  [0.77-0.82] | 0.85  [0.82-0.88] | 0.97  [0.94-1.01] | 0.79  [0.69-0.91] | 1.29  [1.24-1.33] | 1.29  [1.26-1.32] | 0.83  [0.80-0.85] | 0.84  [0.82-0.87] | 1.73  [1.67-1.79] | 1.29  [1.15-1.45] | 0.84  [0.83-0.86] | - |

* The weighted mean is the mean incidence of the variable in which the incidence is weighted for the sample size of each country, so that each country contributes equally to the mean incidence.
