# Supplementary material for: Variations in use of childbirth interventions in 13 high-income countries: A multinational cross-sectional study
Source: PLoS Med. 2020 May 22;17(5):e1003103. doi: 10.1371/journal.pmed.1003103 (PMC7244098; doi:10.1371/journal.pmed.1003103)
Supplement: S3 Table — (DOCX) [file pmed.1003103.s004.docx]

**S3 Table. Crude ORs and adjusted ORs of adverse neonatal and maternal outcomes by country in 2013, compared to the weighted mean, with 99% CIs**

|  | FIN | SWE | NOR | DNK | ISL | IRL | NLD | BEL | HESSE | MLT | USA** | CHL |
| --- | --- | --- | --- | --- | --- | --- | --- | --- | --- | --- | --- | --- |
| Total | 54,310 |  | 54,951 | 50,692 | 3,987 | 62,613 | 152,644 | 112,907 | 44,722 | 3,781 | 3,500,397 | 173,477 |
| Perinatal mortality up to 7 days  Crude OR [99% CI] | 0.91  [0.64-1.29] | 0.92  [0.68-1.24] | 1.01  [0.72-1.43] | 1.11  [0.79-1.56] | 0.53  [0.14-1.96] | - | 1.21  [0.93-1.59] | 1.10  [0.83-1.46] | - | 1.49  [0.66-3.40] | - | - |
| Crude OR [99% CI]  Adjusted * OR [99% CI]  Adjusted # OR [99% CI]  Adjusted^@^ OR [99% CI] | - | - | 1.00  [0.66-1.53]  1.00  [0.66-1.53]  0.99  [0.65-1.50]  1.11  [0.73-1.70] | - | 0.52  [0.16-1.75]  0.52  [0.16-1.75]  0.54  [0.16-1.79]  0.63  [0.19-2.12] | - | 1.19  [0.82-1.74]  1.19  [0.81-1.73]  1.17  [0.80-1.71]  1.16  [0.79-1.70] | 1.08  [0.74-1.60]  1.09  [0.74-1.61]  1.09  [0.74-1.60]  0.98  [0.66-1.44] | - | 1.47  [0.67-3.25]  1.47  [0.66-3.23]  1.49  [0.67-3.29]  1.25  [0.57-2.76] | - | - |
| Crude OR [99% CI]  Adjusted $ OR [99% CI] | - | - | 1.05  [0.64-1.71]  1.04  [0.63-1.70] | - | 0.55  [0.17-1.71]  0.57  [0.18-1.78] | - | - | 1.13  [0.71-1.81]  1.10  [0.69-1.75] | - | 1.54  [0.70-3.37]  1.55  [0.71-3.40] | - | - |
| Crude OR [99% CI]  Adjusted ^ OR [99% CI] | - | - | - | - |  | - | - | 0.86  [0.54-1.37]  0.88  [0.55-1.41] | - | 1.17  [0.73-1.86]  1.14  [0.71-1.82] | - | - |
| Apgar score below 7 at 5 minutes  Crude OR [99% CI] | 1.71  [1.55-1.89] | 1.35  [1.23-1.47] | 0.94  [0.83-1.06] | 0.56  [0.48-0.66] | 1.82  [1.36-2.42] | - | 1.09  [1.00-1.19] | 1.20  [1.09-1.31] | 0.65  [0.56-0.75] | 0.59  [0.36-0.97] | 1.20  [1.13-1.28] | 0.76  [0.69-0.83] |
| Crude OR [99% CI]  Adjusted * OR [99% CI]  Adjusted^@^ OR [99% CI] | - | - | 0.92  [0.80-1.05]  0.93  [0.81-1.07]  0.93  [0.81-1.06] | - | 1.77  [1.34-2.33]  1.81  [1.37-2.39]  1.78  [1.35-2.36] | - | 1.06  [0.95-1.18]  1.06  [0.95-1.18]  1.05  [0.94-1.67] | 1.17  [1.04-1.30]  1.17  [1.05-1.31]  1.18  [1.06-1.32] | - | 0.57  [0.36-0.92]  0.55  [0.34-0.88]  0.55  [0.34-0.89] | 1.17  [1.07-1.29]  1.20  [1.09-1.32]  1.20  [1.09-1.32] | 0.74  [0.66-0.83]  0.73  [0.65-0.81]  0.74  [0.66-0.82] |
| Crude OR [99% CI]  Adjusted # OR [99% CI] | - | - | 0.87  [0.76-1.01]  0.87  [0.75-1.00] | - | 1.68  [1.28-2.21]  1.76  [1.34-2.31] | - | 1.01  [0.90-1.14]  0.98  [0.87-1.11] | 1.11  [0.98-1.25]  1.11  [0.98-1.26] | - | 0.54  [0.34-0.87]  0.52  [0.33-0.83] | 1.12  [1.00-1.24]  1.14  [1.02-1.28] | - |
| Crude OR [99% CI]  Adjusted $ OR [99% CI] | - | - | 0.87  [0.75-1.02]  0.91  [0.78-1.06] | - | 1.69  [1.29-2.21]  1.68  [1.28-2.21] | - | - | 1.11  [0.97-1.28]  1.13  [0.98-1.30] | - | 0.55  [0.35-0.85]  0.53  [0.34-0.82] | 1.12  [0.98-1.27]  1.10  [0.96-1.26] | - |
| Crude OR [99% CI]  Adjusted ^ OR [99% CI] | - | - |  | - |  | - | - | 1.26  [1.05-1.53]  1.29  [1.06-1.56] | - | 0.62  [0.43-0.90]  0.58  [0.40-0.85] | 1.27  [1.06-1.53]  1.33  [1.10-1.60] | - |
| Postpartum haemorrhage ≥1000 ml  Crude OR [99% CI] | **-** | **-** | **-** | **-** | 2.03  [1.69-2.43] | - | 2.46  [2.18-2.77] | - | 0.47  [0.41-0.54] | 0.43  [0.31-0.58] | - | - |
| Crude OR [99% CI]  Adjusted * OR [99% CI]  Adjusted # OR [99% CI]  Adjusted^@^ OR [99% CI] | **-** | **-** | **-** | **-** | 1.58  [1.30-1.91]  1.63  [1.35-1.97]  1.49  [1.23-1.80]  1.50  [1.24-1.82] | - | 1.91  [1.64-2.23]  1.88  [1.61-2.19]  1.95  [1.67-2.27]  1.88  [1.61-2.19] | - | - | 0.33  [0.25-0.44]  0.33  [0.25-0.43]  0.34  [0.26-0.46]  0.35  [0.27-0.47] | - | - |
| Crude OR [99% CI]  Adjusted $ OR [99% CI] | **-** | **-** | **-** | **-** | 2.18  [1.74-2.73]  2.08  [1.65-2.61] | - | - | - | - | 0.46  [0.37-0.57]  0.48  [0.38-0.61] | - | - |
| Obstetric anal sphincter injury in vaginal births  Crude OR [99% CI] | 0.50  [0.45-0.56] | 1.60  [1.51-1.69] | 0.89  [0.81-0.96] | 1.72  [1.60-1.84] | 1.71  [1.38-2.10] | 1.04  [0.96-1.23] | 1.24  [1.17-1.30] | - | 0.67  [0.59-0.75] | - | 0.56  [0.54-0.58] | - |
| Crude OR [99% CI]  Adjusted * OR [99% CI] | - | - | 0.87  [0.80-0.95]  0.84  [0.78-0.92] | - | 1.68  [1.39-2.02]  1.77  [1.47-2.15] | 1.03  [0.94-1.11]  1.01  [0.93-1.10] | 1.22  [1.14-1.30]  1.10  [1.03-1.17] | - | - | - | 0.55  [0.52-0.58]  0.60  [0.57-0.63] | - |
| Crude OR [99% CI]  Adjusted # OR [99% CI] | - | - | 0.88  [0.80-0.96]  0.83  [0.76-0.90] | - | 1.69  [1.41-2.01]  1.70  [1.42-2.02] | - | 1.22  [1.14-1.31]  1.05  [0.98-1.13] | - | - | - | 0.55  [0.52-0.59]  0.68  [0.63-0.73] | - |
| Crude OR [99% CI]  Adjusted $ OR [99% CI] | - | - | 0.94  [0.85-1.03]  0.82  [0.74-0.90] | - | 1.81  [1.54-2.11]  1.75  [1.49-2.05] | - | - | - | - | - | 0.59  [0.54-0.64]  0.70  [0.64-0.77] | - |
| Crude OR [99% CI]  Adjusted^@^ OR [99% CI] | - |  | 0.88  [0.80-0.96]  0.82  [0.75-0.90] | - | 1.69  [1.42-2.01]  1.63  [1.36-1.94] | - | 1.22  [1.14-1.31]  1.13  [1.05-1.22] | - | - | - | 0.55  [0.52-0.59]  0.66  [0.62-0.71] | - |

** Data from USA were randomly compressed ten times in multivariable analyses including ethnicity and education (n=350,040).

* Adjusted for parity and maternal age.

# Adjusted for parity, maternal age and ethnicity.

^@^ Adjusted for parity, maternal age and infant birth weight.

$ Adjusted for parity, maternal age and maternal Body Mass Index.

^ Adjusted for parity, maternal age and education.
