# Supplementary material for: Variations in use of childbirth interventions in 13 high-income countries: A multinational cross-sectional study
Source: PLoS Med. 2020 May 22;17(5):e1003103. doi: 10.1371/journal.pmed.1003103 (PMC7244098; doi:10.1371/journal.pmed.1003103)
Supplement: S4 Table — (DOCX) [file pmed.1003103.s005.docx]

**S4 Table. Crude ORs and for parity and maternal age adjusted ORs of childbirth interventions by country in 2013, compared to the weighted mean, with 99% CIs**

|  | **NOR** | **ISL** | **IRL** | **NLD** | **BEL** | **MLT** | **USA** | **CHL** |
| --- | --- | --- | --- | --- | --- | --- | --- | --- |
| **Total *n*** | 54,951 | 3,987 | 62,613 | 152,644 | 112,907 | 3,781 | 3,500,397 | 173,477 |
| **Spontaneous onset of labour**  Crude OR [99% CI]  Adjusted* OR [99% CI] | 1.91  [1.86-1.97]  1.92  [1.87-1.98] | 1.49  [1.38-1.61]  1.50  [1.39-1.62] | 0.77  [0.75-0.79]  0.81  [0.79-0.83] | 1.05  [1.03-1.08]  1.06  [1.04-1.09] | 0.94  [0.92-0.96]  0.94  [0.92-0.96] | 0.66  [0.62-0.71]  0.65  [0.60-0.70] | 0.69  [0.62-0.71]  0.67  [0.65-0.68] | - |
| **Induction of labour**  Crude OR [99% CI]  Adjusted* OR [99% CI] | 0.62  [0.60-0.64]  0.62  [0.60-0.64] | 0.85  [0.78-0.92]  0.85  [0.78-0.93] | 1.16  [1.13-1.19]  1.20  [1.16-1.23] | 1.19  [1.16-1.22]  1.19  [1.16-1.22] | 1.15  [1.12-1.18]  1.15  [1.12-1.18] | 1.32  [1.22-1.43]  1.28  [1.18-1.39] | 0.91  [0.89-0.93]  0.91  [0.89-0.92] | - |
| **Prelabour CS**  Crude OR [99% CI]  Adjusted* OR [99% CI] | 0.56  [0.53-0.59]  0.55  [0.52-0.58] | 0.53  [0.46-0.62]  0.52  [0.44-0.60] | 1.49  [1.43-1.55]  1.36  [1.30-1.41] | 0.64  [0.61-0.66]  0.62  [0.60-0.65] | 0.97  [0.94-1.01]  0.98  [0.95-1.02] | 1.61  [1.46-1.79]  1.75  [1.58-1.94] | 2.25  [2.18-2.32]  2.42  [2.34-2.49] | - |
| **Augmentation of labour**  Crude OR [99% CI]  Adjusted* OR [99% CI] | 1.82  [1.76-1.88]  1.83  [1.77-1.89] | 0.57  [0.51-0.62]  0.56  [0.51-0.62] | 0.62  [0.60-0.65]  0.64  [0.61-0.66] | 1.08  [1.04-1.11]  1.08  [1.04-1.11] | - | - | 1.42  [1.38-1.45]  1.42  [1.38-1.45] | - |
| **Intrapartum use of oxytocin**  Crude OR [99% CI]  Adjusted* OR [99% CI] | 1.14  [1.10-1.18]  1.15  [1.11-1.20] | 0.64  [0.60-0.68]  0.64  [0.60-0.68] | - | 1.38  [1.33-1.43]  1.36  [1.31-1.41] | - | - | - | - |
| **Artificial rupture of membranes**  Crude OR [99% CI]  Adjusted* OR [99% CI] | - | 1.05  [1.01-1.10]  1.03  [0.99-1.08] | 0.95  [0.91-0.99]  0.97  [0.93-1.01] | - | - | - | - | - |
| **Any pain relief**  Crude OR [99% CI]  Adjusted* OR [99% CI] | 0.94  [0.90-0.98]  0.97  [0.93-1.02] | 1.16  [1.07-1.25]  1.22  [1.13-1.32] | - | 0.30  [0.29-0.31]  0.28  [0.27-0.30] | - | 3.06  [2.67-3.38]  2.97  [2.69-3.29] | - | - |
| **Epidural**  Crude OR [99% CI]  Adjusted* OR [99% CI] | 0.67  [0.65-0.69]  0.67  [0.65-0.69] | 0.92  [0.86-1.00]  0.95  [0.88-1.02] | 1.20  [1.16-1.23]  1.24  [1.21-1.28] | 0.29  [0.29-0.30]  0.28  [0.27-0.29] | 3.10  [3.03-3.18]  3.22  [3.14-3.31] | 0.50  [0.45-0.54]  0.45  [0.41-0.49] | 3.00  [2.94-3.06]  3.14  [3.08-3.21] | - |
| **Other pharmacological pain relief**  Crude OR [99% CI]  Adjusted* OR [99% CI] | 0.93  [0.90-0.97]  0.94  [0.90-0.98] | 0.97  [0.91-1.04]  0.97  [0.91-1.04] | - | 0.27  [0.26-0.28]  0.28  [0.27-0.29] | - | 4.02  [3.70-4.38]  3.96  [3.63-4.31] | - | - |
| **Episiotomy in vaginal births**  Crude OR [99% CI]  Adjusted* OR [99% CI] | 0.72  [0.70-0.75]  0.71  [0.68-0.74] | 0.43  [0.38-0.48]  0.42  [0.37-0.47] | 0.96  [0.92-0.99]  1.02  [0.98-1.06] | 1.19  [1.15-1.23]  1.16  [1.12-1.20] | 2.60  [2.51-2.68]  3.04  [2.93-3.15] | 1.10  [1.00-1.21]  0.95  [0.85-1.05] | - | - |
| **Spontaneous vaginal birth**  Crude OR [99% CI]  Adjusted* OR [99% CI] | 1.39  [1.35-1.43]  1.42  [1.38-1.46] | 1.66  [1.52-1.81]  1.67  [1.53-1.82] | 0.63  [0.61-0.64]  0.67  [0.65-0.68] | 1.48  [1.45-1.51]  1.56  [1.53-1.60] | 1.18  [1.15-1.21]  1.20  [1.17-1.22] | 0.94  [0.87-1.01]  0.96  [0.89-1.04] | 0.93  [0.91-0.94]  0.86  [0.85-0.88] | 0.46  [0.45-0.47]  0.41  [0.40-0.42] |
| **Instrumental vaginal birth**  Crude OR [99% CI]  Adjusted* OR [99% CI] | 1.67  [1.59-1.74]  1.69  [1.62-1.77] | 1.26  [1.10-1.43]  1.31  [1.15-1.50] | 2.60  [2.50-2.71]  2.82  [2.70-2.94] | 1.36  [1.31-1.42]  1.31  [1.26-1.36] | 1.52  [1.46-1.58]  1.50  [1.45-1.57] | 0.74  [0.62-0.87]  0.65  [0.55-0.77] | 0.52  [0.50-0.54]  0.54  [0.52-0.56] | 0.23  [0.22-0.24]  0.23  [0.22-0.24] |
| **Caesarean Section**  Crude OR [99% CI]  Adjusted* OR [99% CI] | 0.57  [0.55-0.59]  0.56  [0.54-0.58] | 0.53  [0.48-0.59]  0.53  [0.47-0.58] | 1.24  [1.21-1.28]  1.14  [1.11-1.17] | 0.60  [0.58-0.61]  0.57  [0.55-0.58] | 0.76  [0.74-0.78]  0.75  [0.74-0.77] | 1.31  [1.20-1.42]  1.30  [1.19-1.41] | 1.41  [1.38-1.44]  1.51  [1.48-1.54] | 3.16  [3.09-3.23]  3.56  [3.48-3.64] |
| **Emergency CS**  Crude OR [99% CI]  Adjusted* OR [99% CI] | 0.90  [0.86-0.94]  0.91  [0.86-0.95] | 0.84  [0.74-0.96]  0.88  [0.77-1.01] | 1.37  [1.31-1.42]  1.43  [1.37-1.49] | 0.88  [0.85-0.91]  0.83  [0.80-0.86] | 0.90  [0.86-0.93]  0.88  [0.85-0.92] | 1.37  [1.23-1.53]  1.23  [1.10-1.38] | 0.90  [0.87-0.92]  0.98  [0.95-1.01] |  |

*Adjusted for parity and maternal age.
