# Supplementary material for: Variations in use of childbirth interventions in 13 high-income countries: A multinational cross-sectional study
Source: PLoS Med. 2020 May 22;17(5):e1003103. doi: 10.1371/journal.pmed.1003103 (PMC7244098; doi:10.1371/journal.pmed.1003103)
Supplement: S5 Table — (DOCX) [file pmed.1003103.s006.docx]

**S5 Table. Crude ORs and for parity, maternal age and ethnicity adjusted ORs of childbirth interventions by country in 2013, compared to the weighted mean, with 99% CIs**

|  | **NOR** | **ISL** | **NLD** | **BEL** | **MLT** | **USA** |
| --- | --- | --- | --- | --- | --- | --- |
| **Total *n*** | 54,951 | 3,987 | 152,644 | 112,907 | 3,781 | 350,040* |
| **Spontaneous onset of labour**  Crude OR [99% CI]  Adjusted # OR [99% CI] | 1.83  [1.78-1.89]  1.83  [1.78-1.89] | 1.43  [1.32-1.54]  1.44  [1.33-1.56] | 1.01  [0.98-1.03]  1.05  [1.03-1.08] | 0.90  [0.88-0.93]  0.90  [0.87-0.92] | 0.64  [0.59-0.68]  0.63  [0.58-0.67] | 0.66  [0.65-0.67]  0.64  [0.62-0.65] |
| **Induction of labour**  Crude OR [99% CI]  Adjusted # OR [99% CI] | 0.63  [0.61-0.65]  0.64  [0.62-0.67] | 0.87  [0.80-0.95]  0.88  [0.80-0.95] | 1.22  [1.19-1.25]  1.20  [1.16-1.23] | 1.19  [1.15-1.21]  1.19  [1.16-1.23] | 1.35  [1.25-1.46]  1.33  [1.23-1.43] | 0.93  [0.91-0.95]  0.94  [0.91-0.96] |
| **Prelabour CS**  Crude OR [99% CI]  Adjusted # OR [99% CI] | 0.60  [0.57-0.63]  0.58  [0.55-0.61] | 0.57  [0.49-0.66]  0.55  [0.47-0.63] | 0.68  [0.65-0.71]  0.65  [0.62-0.68] | 1.04  [1.00-1.08]  1.04  [0.99-1.08] | 1.73  [1.56-1.91]  1.85  [0.67-2.05] | 2.41  [2.32-2.50]  2.56  [2.46-2.65] |
| **Augmentation of labour**  Crude OR [99% CI]  Adjusted # OR [99% CI] | 1.62  [1.56-1.68]  1.99  [1.92-2.06] | 0.50  [0.46-0.55]  0.58  [0.53-0.64] | 0.97  [0.94-1.00]  0.58  [0.57-0.61] | - | - | 1.27  [1.23-1.31]  1.47  [1.43-1.52] |
| **Intrapartum use of oxytocin**  Crude OR [99% CI]  Adjusted # OR [99% CI] | 1.14  [1.10-1.18]  1.18  [1.13-1.22] | 0.64  [0.60-0.68]  0.65  [0.61-0.70] | 1.38  [1.33-1.43]  1.30  [1.25-1.34] | - | - | - |
| **Any pain relief**  Crude OR [99% CI]  Adjusted # OR [99% CI] | 0.94  [0.90-0.98]  0.98  [0.94-1.02] | 1.16  [1.07-1.25]  1.18  [1.09-1.28] | 0.30  [0.29-0.31]  0.24  [0.23-0.25] | - | 3.06  [2.77-3.38]  3.67  [3.32-4.06] | - |
| **Epidural**  Crude OR [99% CI]  Adjusted # OR [99% CI] | 0.69  [0.67-0.71]  0.69  [0.67-0.71] | 0.95  [0.88-1.02]  0.98  [0.91-1.06] | 0.30  [0.29-0.31]  0.29  [0.28-0.29] | 3.20  [3.11-3.28]  3.42  [3.32-3.52] | 0.51  [0.47-0.56]  0.46  [0.42-0.50] | 3.09  [3.02-3.17]  3.30  [3.21-3.39] |
| **Other pharmacological pain relief**  Crude OR [99% CI]  Adjusted # OR [99% CI] | 0.93  [0.90-0.97]  0.99  [0.95-1.02] | 0.97  [0.91-1.04]  0.98  [0.92-1.05] | 0.27  [0.26-0.28]  0.25  [0.24-0.26] | - | 4.02  [3.70-4.38]  4.14  [3.81-4.50] | - |
| **Episiotomy in vaginal births**  Crude OR [99% CI]  Adjusted # OR [99% CI] | 0.72  [0.69-0.75]  0.62  [0.60-0.65] | 0.42  [0.38-0.47]  0.37  [0.33-0.42] | 1.18  [1.14-1.23]  1.38  [1.33-1.43] | 2.57  [2.48-2.67]  3.39  [3.27-3.53] | 1.09  [0.99-1.20]  0.93  [0.85-1.01] | - |
| **Spontaneous vaginal birth**  Crude OR [99% CI]  Adjusted # OR [99% CI] | 1.13  [1.09-1.16]  1.15  [1.11-1.19] | 1.35  [1.24-1.46]  1.31  [1.21-1.43] | 1.20  [1.17-1.23]  1.29  [1.26-1.32] | 0.96  [0.93-0.98]  0.96  [0.94-0.99] | 0.76  [0.70-0.82]  0.78  [0.72-0.84] | 0.75  [0.74-0.77]  0.68  [0.67-0.70] |
| **Instrumental vaginal birth**  Crude OR [99% CI]  Adjusted # OR [99% CI] | 1.53  [1.45-1.61]  1.58  [1.50-1.67] | 1.15  [1.01-1.31]  1.24  [1.09-1.42] | 1.25  [1.20-1.31]  1.19  [1.14-1.25] | 1.40  [1.33-1.46]  1.40  [1.34-1.47] | 0.68  [0.57-0.80]  0.59  [0.50-0.70] | 0.48  [0.46-0.50]  0.51  [0.49-0.54] |
| **Caesarean Section**  Crude OR [99% CI]  Adjusted # OR [99% CI] | 0.72  [0.69-0.74]  0.70  [0.67-0.72] | 0.67  [0.61-0.74]  0.68  [0.61-0.75] | 0.75  [0.73-0.77]  0.70  [0.68-0.73] | 0.96  [0.93-0.99]  0.95  [0.92-0.98] | 1.64  [1.52-1.78]  1.65  [1.52-1.79] | 1.77  [1.73-1.82]  1.92  [1.86-1.97] |
| **Emergency CS**  Crude OR [99% CI]  Adjusted # OR [99% CI] | 0.95  [0.91-0.99]  0.94  [0.90-0.99] | 0.89  [0.78-1.00]  0.95  [0.84-1.08] | 0.93  [0.89-0.96]  0.86  [0.83-0.90] | 0.94  [0.91-0.98]  0.94  [0.90-0.98] | 1.44  [1.29-1.61]  1.34  [1.20-1.50] | 0.94  [0.91-0.98]  1.03  [0.99-1.07] |

*Data from USA were randomly compressed ten times in multivariable analyses including ethnicity, BMI, and education.

# Adjusted for parity, maternal age and ethnicity.
