# Supplementary material for: Variations in use of childbirth interventions in 13 high-income countries: A multinational cross-sectional study
Source: PLoS Med. 2020 May 22;17(5):e1003103. doi: 10.1371/journal.pmed.1003103 (PMC7244098; doi:10.1371/journal.pmed.1003103)
Supplement: S6 Table — (DOCX) [file pmed.1003103.s007.docx]

**S6 Table. Crude ORs and for parity, maternal age and maternal Body Mass Index adjusted ORs of childbirth interventions by country in 2013, compared to the weighted
mean, with 99% CIs**

|  | **NOR** | **ISL** | **BEL** | **MLT** | **USA** |
| --- | --- | --- | --- | --- | --- |
| **Total *n*** | 54,951 | 3,987 | 112,907 | 3,781 | 350,040* |
| **Spontaneous onset of labour**  Crude OR [99% CI]  Adjusted $ OR [99% CI] | 1.83  [1.78-1.90]  1.77  [1.71-1.83] | 1.43  [1.33-1.54]  1.53  [1.42-1.65] | 0.90  [0.88-0.93]  0.88  [0.85-0.91] | 0.64  [0.59-0.68]  0.61  [0.57-0.65] | 0.66  [0.64-0.68]  0.69  [0.67-0.71] |
| **Induction of labour**  Crude OR [99% CI]  Adjusted $ OR [99% CI] | 0.66  [0.63-0.68]  0.68  [0.66-0.71] | 0.91  [0.83-0.98]  0.89  [0.82-0.96] | 1.23  [1.19-1.27]  1.26  [1.22-1.30] | 1.41  [1.31-1.52]  1.41  [1.30-1.52] | 0.97  [0.94-1.00]  0.93  [0.91-0.96] |
| **Prelabour CS**  Crude OR [99% CI]  Adjusted $ OR [99% CI] | 0.55  [0.52-0.59]  0.56  [0.53-0.60] | 0.53  [0.46-0.61]  0.48  [0.41-0.55] | 0.96  [0.92-1.01]  0.98  [0.93-1.02] | 1.60  [1.45-1.76]  1.75  [1.58-1.93] | 2.23  [2.13-2.33]  2.20  [2.10-2.30] |
| **Augmentation of labour**  Crude OR [99% CI]  Adjusted $ OR [99% CI] | 1.60  [1.53-1.67]  1.71  [1.64-1.79] | 0.50  [0.46-0.54]  0.48  [0.44-0.52] | - | - | 1.26  [1.21-1.31]  1.23  [1.18-1.28] |
| **Intrapartum use of oxytocin**  Crude OR [99% CI]  Adjusted $ OR [99% CI] | 1.34  [1.27-1.40]  1.40  [1.33-1.47] | 0.75  [0.71-0.79]  0.71  [0.68-0.75] | - | - | - |
| **Any pain relief**  Crude OR [99% CI]  Adjusted $ OR [99% CI] | 0.63  [0.60-0.67]  0.63  [0.60-0.67] | 0.77  [0.72-0.83]  0.70  [0.64-0.75] | - | 2.05  [1.87-2.24]  2.28  [2.08-2.50] | - |
| **Epidural**  Crude OR [99% CI]  Adjusted $ OR [99% CI] | 0.54  [0.52-0.56]  0.54  [0.53-0.57] | 0.75  [0.70-0.80]  0.75  [0.70-0.81] | 2.52  [2.44-2.59]  2.66  [2.58-2.75] | 0.40  [0.37-0.44]  0.36  [0.33-0.40] | 2.43  [2.37-2.50]  2.51  [2.44-2.59] |
| **Other pharmacological pain relief**  Crude OR [99% CI]  Adjusted $ OR [99% CI] | 0.61  [0.58-0.64]  0.63  [0.60-0.66] | 0.63  [0.59-0.68]  0.60  [0.58-0.64] | - | 2.62  [2.42-2.72]  2.65  [2.45-2.86] | - |
| **Episiotomy in vaginal births**  Crude OR [99% CI]  Adjusted $ OR [99% CI] | 0.75  [0.71-0.79]  0.65  [0.62-0.69] | 0.44  [0.40-0.49]  0.42  [0.37-0.46] | 2.68  [2.56-2.81]  3.65  [3.49-3.83] | 1.13  [1.04-1.24]  1.01  [0.92-1.10] | - |
| **Spontaneous vaginal birth**  Crude OR [99% CI]  Adjusted $ OR [99% CI] | 1.17  [1.13-1.21]  1.13  [1.09-1.17] | 1.40  [1.29-1.51]  1.47  [1.35-1.59] | 0.99  [0.96-1.02]  0.98  [0.95-1.01] | 0.79  [0.73-0.85]  0.80  [0.74-0.86] | 0.78  [0.76-0.80]  0.77  [0.75-0.79] |
| **Instrumental vaginal birth**  Crude OR [99% CI]  Adjusted $ OR [99% CI] | 1.60  [1.51-1.70]  1.62  [1.52-1.71] | 1.21  [1.06-1.37]  1.29  [1.14-1.47] | 1.46  [1.38-1.54]  1.44  [1.37-1.52] | 0.71  [0.60-0.83]  0.61  [0.52-0.72] | 0.50  [0.48-0.53]  0.54  [0.51-0.57] |
| **Caesarean Section**  Crude OR [99% CI]  Adjusted $ OR [99% CI] | 0.67  [0.65-0.70]  0.70  [0.67-0.73] | 0.63  [0.57-0.70]  0.58  [0.53-0.64] | 0.90  [0.87-0.94]  0.92  [0.89-0.95] | 1.55  [1.43-1.68]  1.66  [1.61-1.72] | 1.67  [1.62-1.73]  1.66  [1.61-1.72] |
| **Emergency CS**  Crude OR [99% CI]  Adjusted $ OR [99% CI] | 0.93  [0.89-0.98]  0.99  [0.94-1.04] | 0.87  [0.77-0.96]  0.86  [0.76-0.97] | 0.93  [0.89-0.97]  0.94  [0.90-0.99] | 1.42  [1.28-1.58]  1.33  [1.19-1.48] | 0.93  [0.89-0.97]  0.94  [0.90-0.98] |

*Data from USA were randomly compressed ten times in multivariable analyses including ethnicity, BMI, and education.

$ Adjusted for parity, maternal age and maternal Body Mass Index.
