# Supplementary material for: Variations in use of childbirth interventions in 13 high-income countries: A multinational cross-sectional study
Source: PLoS Med. 2020 May 22;17(5):e1003103. doi: 10.1371/journal.pmed.1003103 (PMC7244098; doi:10.1371/journal.pmed.1003103)
Supplement: S7 Table — (DOCX) [file pmed.1003103.s008.docx]

**S7 Table. Crude ORs and for parity, maternal age and education adjusted ORs of childbirth interventions by country in 2013, compared to the weighted mean, with 99% CIs**

|  | **BEL** | **MLT** | **USA** | **CHL** |
| --- | --- | --- | --- | --- |
| **Total *n*** | 112,907 | 3,781 | 350,040* | 173,477 |
| **Spontaneous onset of labour**  Crude OR [99% CI]  Adjusted^ OR [99% CI] | 1.25  [1.21-1.28]  1.25  [1.21-1.29] | 0.88  [0.83-0.93]  0.89  [0.84-0.94] | 0.91  [0.89-0.94]  0.90  [0.87-0.92] | - |
| **Induction of labour**  Crude OR [99% CI]  Adjusted^ OR [99% CI] | 1.03  [1.00-1.07]  1.06  [1.02-1.09] | 1.19  [1.12-1.26]  1.15  [1.07-1.22] | 0.82  [0.79-0.84]  0.82  [0.80-0.85] | - |
| **Prelabour CS**  Crude OR [99% CI]  Adjusted^ OR [99% CI] | 0.64  [0.61-0.67]  0.62  [0.59-0.64] | 1.06  [0.98-1.14]  1.08  [1.00-1.16] | 1.48  [1.42-1.54]  1.51  [1.45-1.57] | - |
| **Epidural**  Crude OR [99% CI]  Adjusted^ OR [99% CI] | 1.86  [1.80-1.93]  2.00  [1.92-2.07] | 0.30  [0.28-0.32]  0.26  [0.24-0.28] | 1.80  [1.74-1.87]  1.91  [1.83-1.98] | - |
| **Episiotomy in vaginal births**  Crude OR [99% CI]  Adjusted^ OR [99% CI] | 1.54  [1.45-1.63]  1.90  [1.79-2.00] | 0.65  [0.61-0.69]  0.53  [0.50-0.56] | - | - |
| **Spontaneous vaginal birth**  Crude OR [99% CI]  Adjusted^ OR [99% CI] | 1.42  [1.39-1.46]  1.52  [1.48-1.56] | 1.13  [1.06-1.21]  1.24  [1.15-1.32] | 1.12  [1.10-1.15]  1.07  [1.05-1.10] | 0.55  [0.54-0.57]  0.50  [0.49-0.51] |
| **Instrumental vaginal birth**  Crude OR [99% CI]  Adjusted^ OR [99% CI] | 2.50  [2.37-2.64]  2.55  [2.41-2.70] | 1.21  [1.05-1.40]  1.09  [0.94-1.27] | 0.86  [0.82-0.91]  0.91  [0.87-0.97] | 0.38  [0.36-0.41]  0.39  [0.37-0.42] |
| **Caesarean Section**  Crude OR [99% CI]  Adjusted^ OR [99% CI] | 0.53  [0.51-0.54]  0.49  [0.48-0.51] | 0.90  [0.84-0.97]  0.84  [0.78-0.90] | 0.97  [0.95-1.00]  1.01  [0.98-1.03] | 2.17  [2.12-2.23]  2.40  [2.34-2.46] |
| **Emergency CS**  Crude OR [99% CI]  Adjusted^ OR [99% CI] | 0.87  [0.83-0.91]  0.87  [0.83-0.91] | 1.33  [1.22-1.44]  1.18  [1.08-1.29] | 0.87  [0.83-0.91]  0.97  [0.93-1.02] | - |

*Data from USA were randomly compressed ten times in multivariable analyses including ethnicity and education.

^Adjusted for parity, maternal age and education.
