# Supplementary material for: Variations in use of childbirth interventions in 13 high-income countries: A multinational cross-sectional study
Source: PLoS Med. 2020 May 22;17(5):e1003103. doi: 10.1371/journal.pmed.1003103 (PMC7244098; doi:10.1371/journal.pmed.1003103)
Supplement: S8 Table — (DOCX) [file pmed.1003103.s009.docx]

**S8 Table. Crude ORs and for parity, maternal age and birth weight adjusted ORs of childbirth interventions by country in 2013, compared to the weighted mean, with 99% CIs**

|  | **NOR** | **ISL** | **NLD** | **BEL** | **MLT** | **USA** | **CHL** |
| --- | --- | --- | --- | --- | --- | --- | --- |
| **Total *n*** | 54,951 | 3,987 | 152,644 | 112,907 | 3,781 | 3,500,397 | 173,477 |
| **Spontaneous onset of labour**  Crude OR [99% CI]  Adjusted # OR [99% CI] | 1.83  [1.78-1.89]  1.90  [1.84-1.95] | 1.43  [1.32-1.54]  1.51  [1.40-1.63] | 1.01  [0.98-1.03]  1.03  [1.00-1.05] | 0.90  [0.88-0.93]  0.89  [0.86-0.91] | 0.64  [0.59-0.68]  0.61  [0.56-0.65] | 0.66  [0.65-0.67]  0.63  [0.62-0.65] | - |
| **Induction of labour**  Crude OR [99% CI]  Adjusted # OR [99% CI] | 0.63  [0.61-0.65]  0.62  [0.60-0.65] | 0.87  [0.80-0.95]  0.85  [0.79-0.93] | 1.22  [1.19-1.25]  1.22  [1.19-1.25] | 1.18  [1.15-1.21]  1.20  [1.17-1.23] | 1.35  [1.25-1.46]  1.36  [1.25-1.47] | 0.93  [0.91-0.95]  0.94  [0.92-0.97] | - |
| **Prelabour CS**  Crude OR [99% CI]  Adjusted # OR [99% CI] | 0.60  [0.57-0.63]  0.57  [0.54-0.60] | 0.57  [0.49-0.66]  0.52  [0.45-0.61] | 0.68  [0.65-0.71]  0.66  [0.63-0.68] | 1.04  [1.00-1.08]  1.05  [1.01-1.10] | 1.73  [1.56-1.91]  1.88  [1.70-2.09] | 2.41  [2.32-2.50]  2.58  [2.48-2.67] | - |
| **Augmentation of labour**  Crude OR [99% CI]  Adjusted # OR [99% CI] | 1.62  [1.56-1.68]  1.63  [1.57-1.69] | 0.50  [0.46-0.55]  0.50  [0.46-0.55] | 0.97  [0.94-1.00]  0.96  [0.96-1.00] | - | - | 1.27  [1.23-1.31]  1.28  [1.24-1.32] | - |
| **Intrapartum use of oxytocin**  Crude OR [99% CI]  Adjusted # OR [99% CI] | 1.14  [1.10-1.18]  1.16  [1.11-1.20] | 0.64  [0.60-0.68]  0.63  [0.58-0.67] | 1.38  [1.33-1.43]  1.38  [1.33-1.43] | - | - | - | - |
| **Any pain relief**  Crude OR [99% CI]  Adjusted^@^ OR [99% CI] | 0.94  [0.90-0.98]  0.96  [0.92-1.01] | 1.16  [1.07-1.25]  1.19  [1.10-1.29] | 0.30  [0.29-0.31]  0.28  [0.27-0.30] | 3.06  [2.77-3.38]  3.06  [2.77-3.39] | - | - | - |
| **Epidural**  Crude OR [99% CI]  Adjusted^@^ OR [99% CI] | 0.69  [0.67-0.71]  0.69  [0.67-0.71] | 0.95  [0.88-1.02]  0.97  [0.90-1.04] | 0.30  [0.29-0.31]  0.29  [0.28-0.30] | 3.20  [3.11-3.28]  3.36  [3.27-3.45] | 0.51  [0.47-0.56]  0.47  [0.43-0.51] | 3.09  [3.02-3.17]  3.28  [3.20-3.36] | - |
| **Other pharmacological pain relief**  Crude OR [99% CI]  Adjusted* OR [99% CI] | 0.93  [0.90-0.97]  0.93  [0.90-0.97] | 0.97  [0.91-1.04]  0.96  [0.90-1.03] | 0.27  [0.26-0.28]  0.28  [0.27-0.29] | - | 4.02  [3.70-4.38]  4.03  [3.70-4.38] | - | - |
| **Episiotomy in vaginal births**  Crude OR [99% CI]  Adjusted^@^ OR [99% CI] | 0.72  [0.69-0.75]  0.69  [0.66-0.72] | 0.42  [0.38-0.47]  0.40  [0.36-0.45] | 1.18  [1.14-1.23]  1.16  [1.12-1.21] | 2.57  [2.48-2.67]  3.12  [2.99-3.25] | 1.09  [0.99-1.20]  1.00  [0.90-1.10] | - | - |
| **Spontaneous vaginal birth**  Crude OR [99% CI]  Adjusted^@^ OR [99% CI] | 1.30  [1.26-1.34]  1.37  [1.33-1.41] | 1.55  [1.42-1.69]  1.66  [1.52-1.81] | 1.38  [1.35-1.42]  1.48  [1.45-1.52] | 1.10  [1.08-1.13]  1.11  [1.08-1.13] | 0.88  [0.81-0.95]  0.88  [0.81-0.95] | 0.87  [0.85-0.88]  0.80  [0.79-0.82] | 0.43  [0.42-0.44]  0.38  [0.37-0.39] |
| **Instrumental vaginal birth**  Crude OR [99% CI]  Adjusted^@^ OR [99% CI] | 1.91  [1.82-2.00]  1.96  [1.86-2.05] | 1.44  [1.26-1.64]  1.52  [1.33-1.73] | 1.56  [1.50-1.63]  1.52  [1.46-1.59] | 1.74  [1.67-1.82]  1.75  [1.67-1.82] | 0.84  [0.71-1.00]  0.76  [0.64-0.90] | 0.60  [0.58-0.62]  0.63  [0.60-0.65] | 0.27  [0.25-0.28]  0.27  [0.25-0.28] |
| **Caesarean Section**  Crude OR [99% CI]  Adjusted^@^ OR [99% CI] | 0.59  [0.57-0.61]  0.55  [0.53-0.57] | 0.55  [0.50-0.61]  0.51  [0.46-0.56] | 0.61  [0.60-0.63]  0.57  [0.56-0.59] | 0.79  [0.77-0.81]  0.78  [0.76-0.81] | 1.35  [1.24-1.46]  1.37  [1.26-1.49] | 1.46  [1.42-1.49]  1.57  [1.53-1.60] | 3.26  [3.18-3.34]  3.71  [3.62-3.80] |
| **Emergency CS**  Crude OR [99% CI]  Adjusted^@^ OR [99% CI] | 0.95  [0.91-0.99]  0.93  [0.88-0.97] | 0.89  [0.78-1.00]  0.87  [0.76-0.99] | 0.93  [0.89-0.96]  0.87  [0.84-0.91] | 0.94  [0.91-0.98]  0.97  [0.93-1.01] | 1.44  [1.29-1.61]  1.38  [1.24-1.55] | 0.94  [0.93-0.98]  1.06  [1.02-1.10] | - |

^@^ Adjusted for parity, maternal age and infant birth weight.
