# Supplementary material for: Variations in use of childbirth interventions in 13 high-income countries: A multinational cross-sectional study
Source: PLoS Med. 2020 May 22;17(5):e1003103. doi: 10.1371/journal.pmed.1003103 (PMC7244098; doi:10.1371/journal.pmed.1003103)
Supplement: S1 Data Statement — (PDF) [file pmed.1003103.s010.pdf]

Supplementary information

Details on data statements per country

Finland:
"Finnish data were taken from the Medical Birth Register as instructed by the research group and delivered in aggregated format. Researchers interested in inquiring about access to data may do so via tietopyynnot@thl.fi. Similar statistical data can be requested from this e-mail address. Also the right to use anonymized register data can be applied. More information on the authorization application to researchers who meet the criteria for access to confidential data can be found at https://thl.fi/fi/web/thlfi-en/statistics/information-for-researchers/authorisation-application)."

Sweden:
"Swedish data are available on group and sub-group level at the website for the National board of health and welfare (sos.se). Researchers interested in inquiring about access to data may do so via registerservice@socialstyrelsen.se (https://etikprovningsmyndigheten.se/). Data on individuals are available from the National board of health and welfare for researchers who apply for and receive permission from the Swedish Ethical Review Authority."

Norway:
"Norwegian data from cannot be shared publicly because of restrictions of the Norwegian health research act §7 and §17, and the Medical Birth Registry of Norway §1-3 and §3-5. Researchers interested in inquiring about access to data may do so via: https://www.fhi.no/en/op/data-access-from-health-registries-health-studies-and-biobanks/medical-birth-registry-and-registry-of-pregnancy-termination/access-to-data/."

Denmark:
"Danish data cannot be shared publicly because of restrictions on the register. According to Danish legislation, data used in this study can only be made available through a trusted third party, i.e. Statistics Denmark. Researchers interested in inquiring about access to data may do so via way.dst@dst.dk."

Iceland:
"Icelandic data cannot be shared publicly because of restrictions of the Icelandic Birth Registration. Researchers interested in inquiring about access to data may do so via the Directorate of Health (contact via mottaka@landlaeknir.is), for researchers who apply for and receive permission from the Directorate of Health (https://www.landlaeknir.is), the National Bioethics Committee (https://www.vsn.is) and (if applicable) the Data Protection Authority (https://www.personuvernd.is/)."

Ireland:
"Irish data cannot be shared publicly because of restrictions in the Conditions of Use Agreement. Researchers interested in inquiring about access to data may do so via the Healthcare Pricing Office: info@hpo.ie."

England:
“English data cannot be shared publicly because of restrictions of NHS Digital. Researchers interested in inquiring about access to data may do so via NHS Digital: hes.questions@nhs.net. NHS Digital should give permission.”

The Netherlands:
“Dutch data cannot be shared publicly because of restrictions of the perinatal register "Perined". Researchers interested in inquiring about access to data may do so via info@perined.nl. Data are available from Perined for researchers who meet the criteria for access to confidential data, and if Perined gives permission.”

Belgium:
“Belgian data cannot be shared publicly because of restrictions of the “eBirth Medical” and “eBirth City”. Researchers interested in inquiring about access to data may do so via servicedesk.DTO@bosa.fgov.be or https://dt.bosa.be/nl/gegevensuitwisseling/combinatie_van_gegevens/ebirth. Data can be made available for researchers after fulfilling strict criteria by an authorization request.”

The state of Hesse (Germany):
“Data from the state of Hesse, which were used for the purpose of this research, were carried out from a dataset of a national wide Quality assurance program. Researchers interested in inquiring about access to data may do so via the Institute of Quality Assurance Hesse: mail@gqhnet.de.”

Malta:
“Maltese data were taken from the National Obstetric Information System (NOIS) which covers all the births happening in all the hospitals in Malta and the sister island Gozo. Researchers interested in inquiring about access to data may do so via healthinfo@gov.mt or https://deputyprimeminister.gov.mt/en/dhir/Pages/request-for-data-form.aspx.”

United States:
“USA data were all from publicly available sources. Specifically, the data was from the free publicly downloadable datasets available at: https://www.cdc.gov/nchs/data_access/vitalstatsonline.htm. For a more accessible form of U.S. data one can go to CDC Wonder (https://wonder.cdc.gov/natality.html) which has data going back to 1995, but is most complete for data from 2016 onward.”

Chile:
“Chilean data cannot be shared publicly because of restrictions of The Ministry of Health Chile. Researchers interested in inquiring about access to data may do so via the Department of Statistics and Health Information, Data Base of Birth: http://www.deis.cl/bases-de-datos-nacimientos/; and Hospital Discharges: http://www.deis.cl/bases-de-datos-egresos-hospitalarios/.”
