# Supplementary figures and images for: Variations in use of childbirth interventions in 13 high-income countries: A multinational cross-sectional study
Source: PLoS Med. 2020 May 22;17(5):e1003103. doi: 10.1371/journal.pmed.1003103 (PMC7244098; doi:10.1371/journal.pmed.1003103)

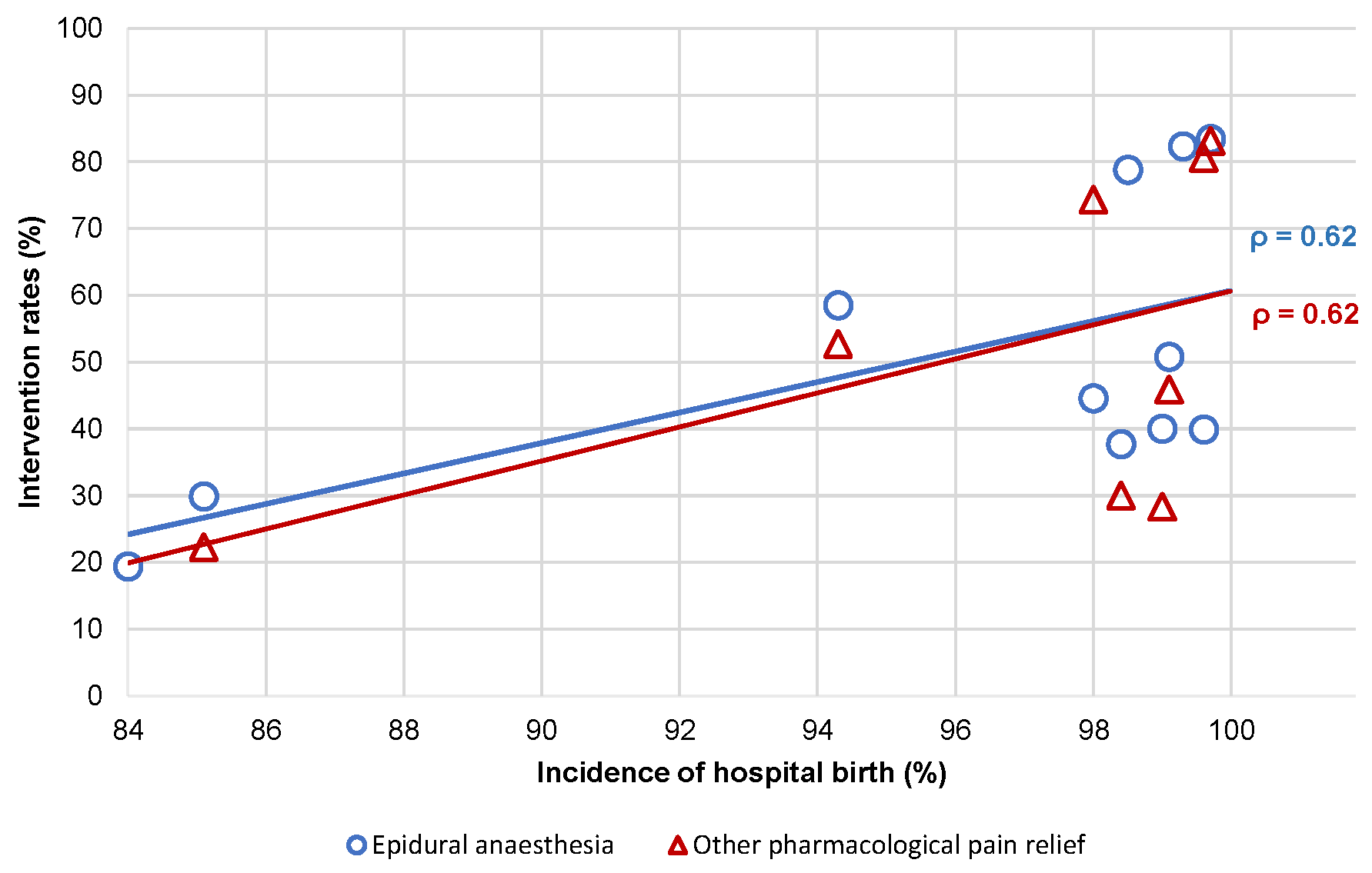

Supplement: S1 Fig — (TIF) [file pmed.1003103.s011.tif]

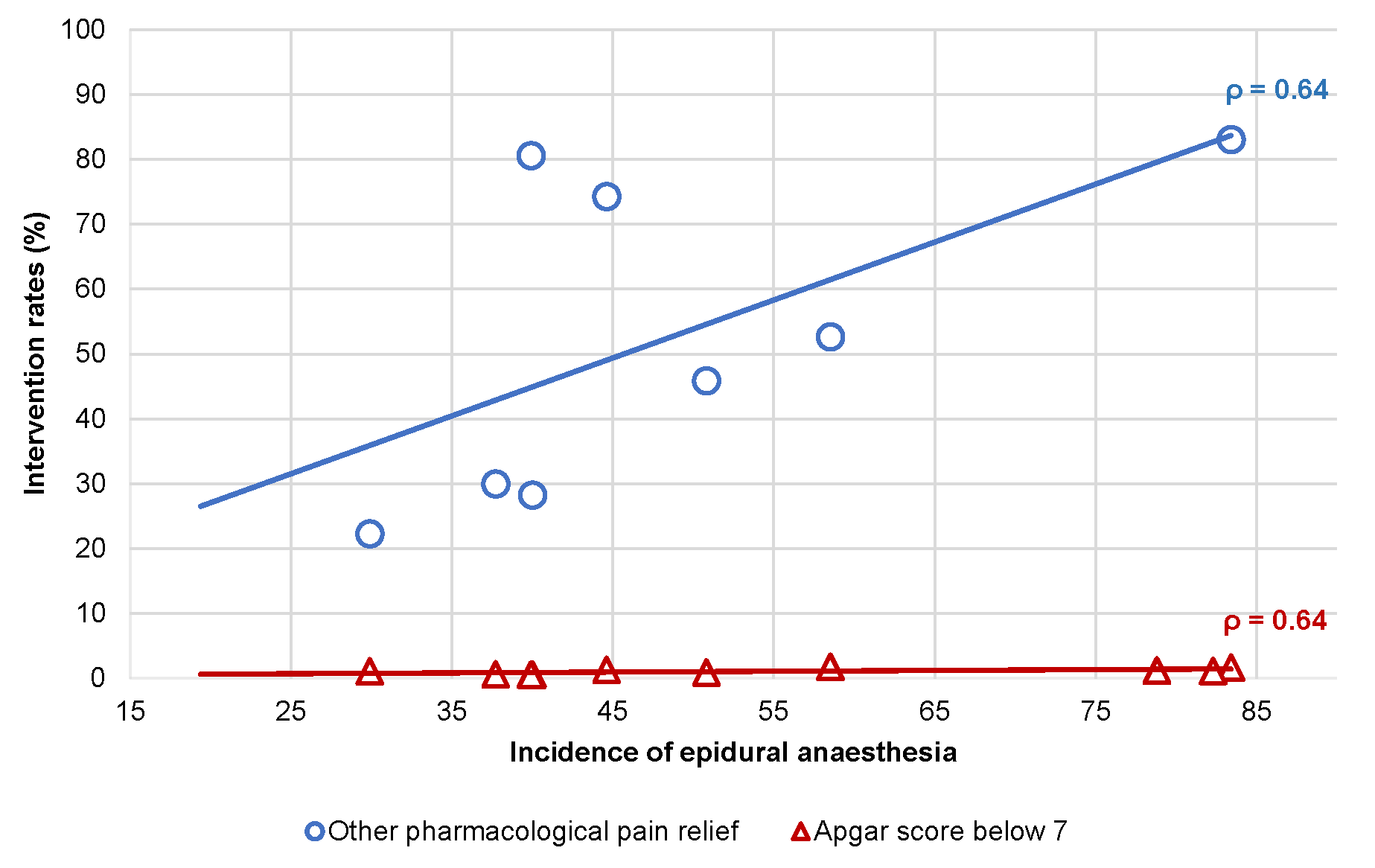

Supplement: S2 Fig — (TIF) [file pmed.1003103.s012.tif]

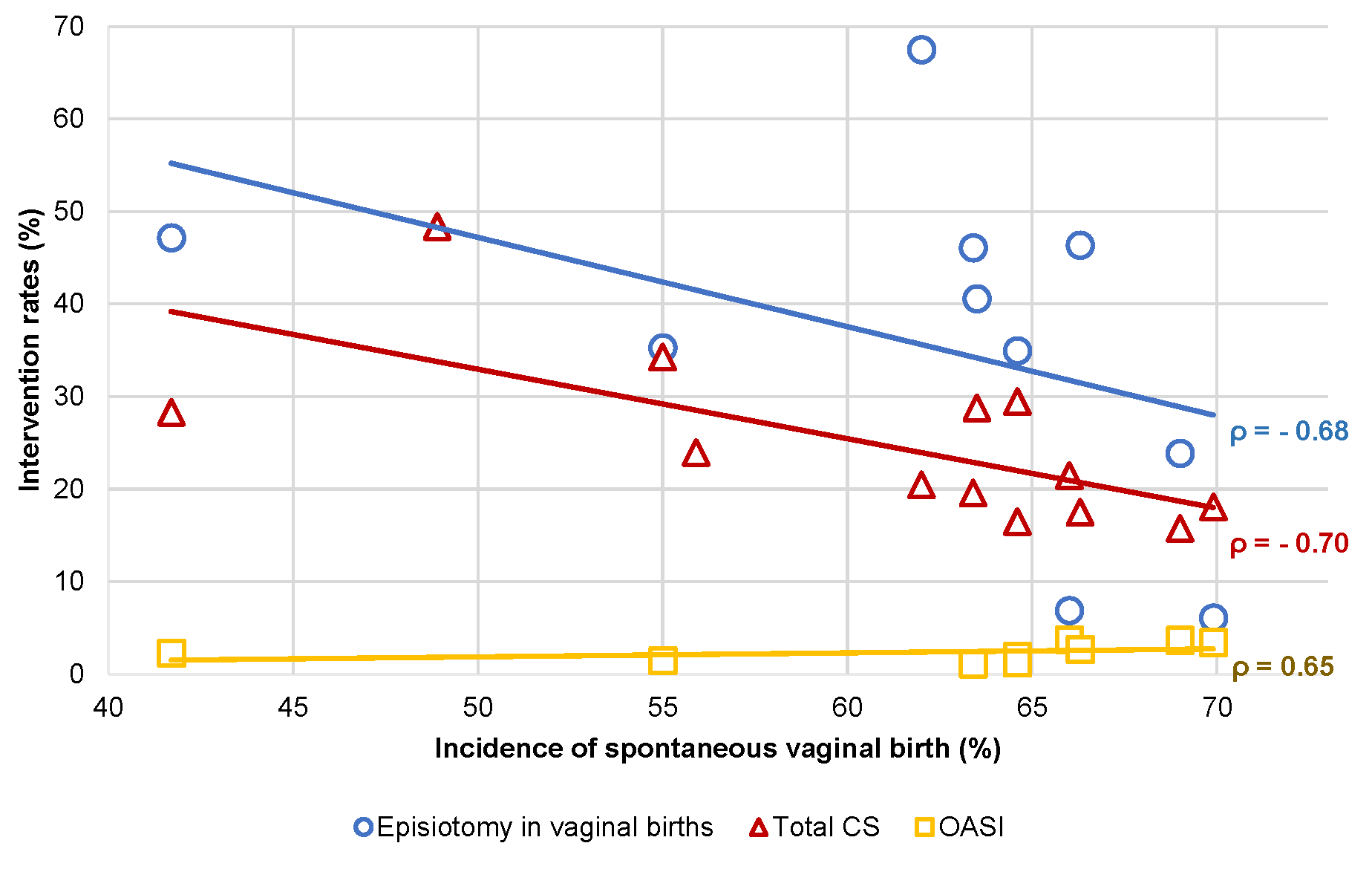

Supplement: S3 Fig — (TIF) [file pmed.1003103.s013.tif]

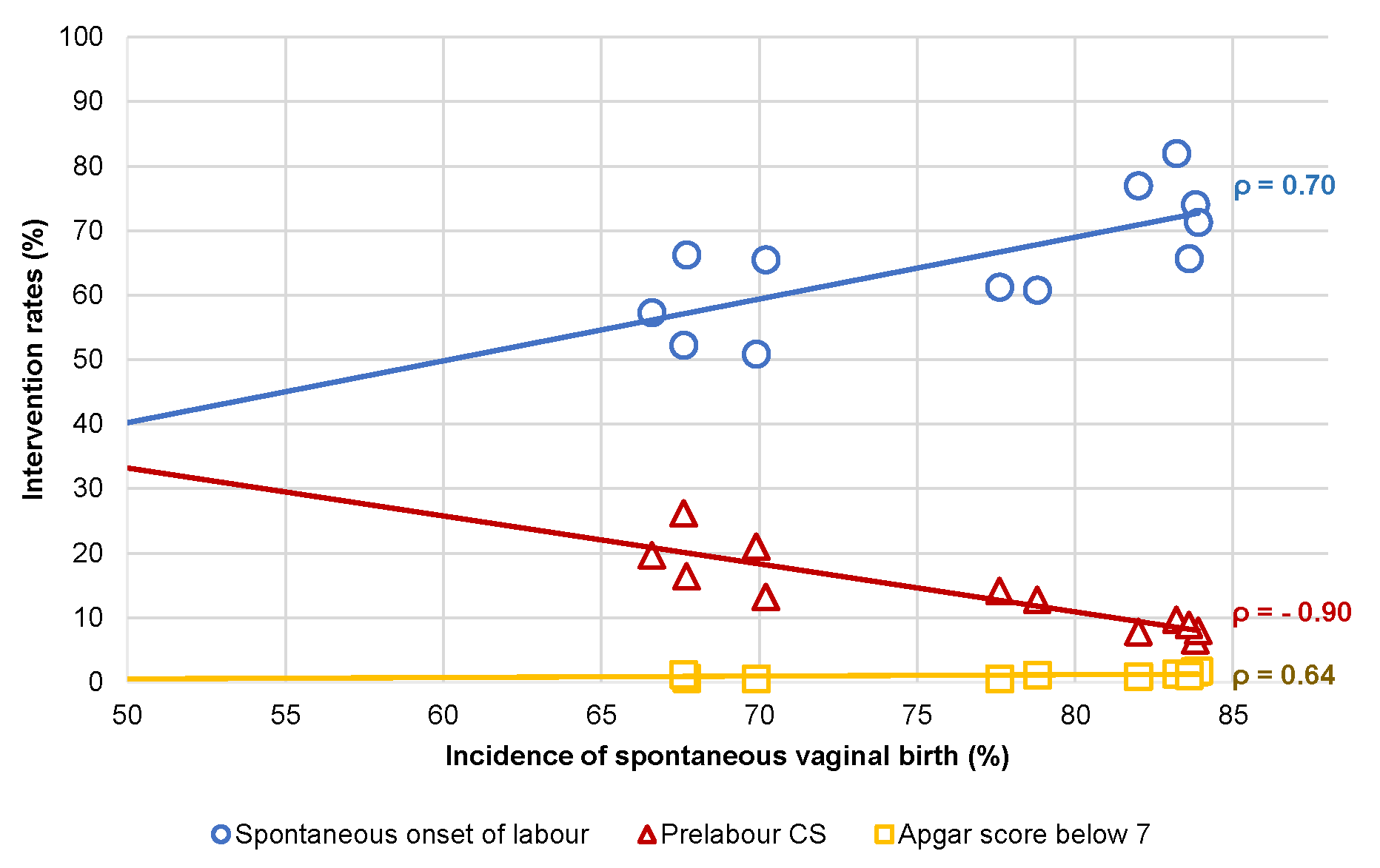

Supplement: S4 Fig — (TIF) [file pmed.1003103.s014.tif]
